# Discovery of molybdenum based nitrogen fixation catalysts with genetic algorithms†

**DOI:** 10.1039/d4sc02227k

**Published:** 2024-06-07

**Authors:** Magnus Strandgaard, Julius Seumer, Jan H. Jensen

**Affiliations:** a Department of Chemistry, University of Copenhagen Denmark jhjensen@chem.ku.dk https://twitter.com/janhjensen

## Abstract

Computational discovery of organometallic catalysts that effectively catalyze nitrogen fixation is a difficult task. The complexity of the chemical reactions involved and the lack of understanding of natures enzyme catalysts raises the need for intricate computational models. In this study, we use a dataset of 91 experimentally verified ligands as starting population for a Genetic Algorithm (GA) and use this to discover molybdenum based nitrogen fixation catalyst in trigonal bipyramidal and octahedral configurations. Through evolutionary discovery with a semi-empirical quantum method driven GA and a density functional theory (DFT) based screening process, we find 3 promising catalyst candidates that are shown to effectively catalyze the first protonation step of the Schrock cycle. Synthetic accessibility (SA) scores are used to guide the GA towards reasonable ligands and the work features a description of the GA framework, including pre-screening of catalyst candidates that involves assignment of metal coordination atoms and catalyst stereoisomers. This research thus not only offers insights into the specific field of molybdenum-based catalysts for nitrogen fixation but also demonstrates the broader applicability and potential of genetic algorithms in the field of catalyst discovery and materials science.

## Abstract 2

Using genetic algorithms and semiempirical quantum mechanical methods for discovery of nitrogen fixation catalysts.

## Graphical abstract

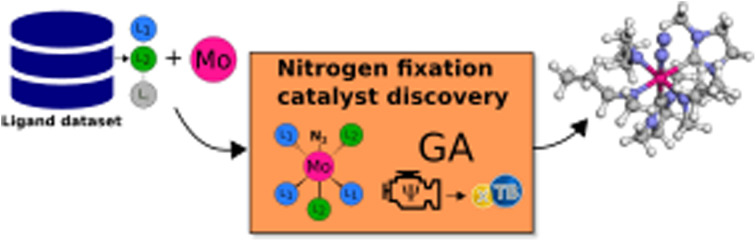

## 1. Introduction

**Fig. 1:**
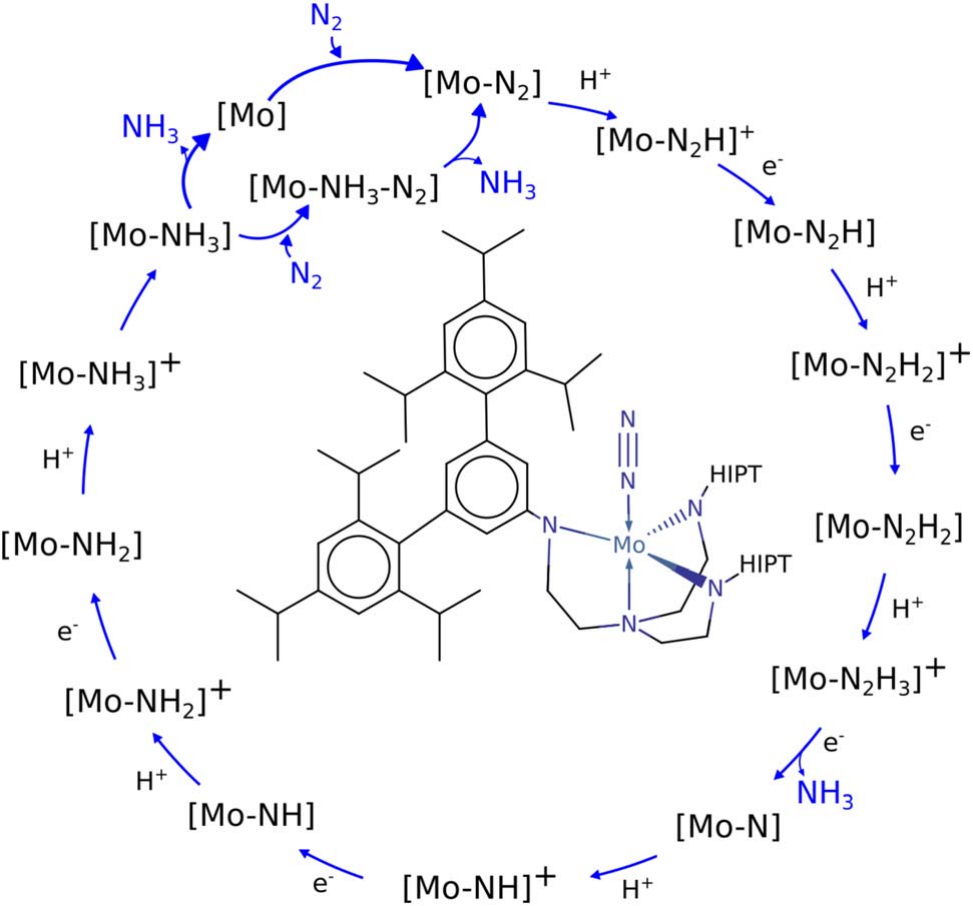
Schematic of the Schrock cycle with the Schrock catalyst in the middle.^24^

Nitrogen fixation is a cornerstone of modern agriculture and industrial chemistry and revolves around the conversion of atmospheric nitrogen N_2_ into ammonia NH_3_. Plants perform nitrogen fixation to produce nitrides for their own sustained survival through the intricate and complex mechanisms of nitrogenases. These enzyme catalysts are based on variations of an iron based cofactor combined with molybdenum or vanadium and we are yet to understand these mechanisms and replicate them in grand-scale ammonia synthesis.^1,2^ The current industrial standard for nitrogen fixation, the Haber–Bosch process, while effective, demands high energy inputs and operates under extreme conditions. In light of this, substantial effort has been made to search for more sustainable methods. Advances have been made within homogeneous and heterogeneous catalysis as well as photo- and electrocatalytic catalysis to address this challenge.^3^ Within efforts made towards finding homogeneous catalysts, transition metal complexes (TMCs) have shown significant promise due to their unique electronic properties and ability to coordinate with nitrogen molecules, and they offer a promising avenue for catalyzing nitrogen fixation under milder conditions. Particularly complexes involving molybdenum, iron, cobalt, and vanadium, have been identified as effective catalysts, offering insights into the mechanisms of nitrogen fixation and the design of more efficient catalytic systems. Molybdenum complexes have seen the most success so far with different types of reactions. Examples include homogenous catalysis *via* bridged dinitrogen compounds^4^ or direct cleavage of the dinitrogen in Mo–N_2_–Mo compounds.^5–7^ One of the most well known examples is the Schrock catalyst ([Mo(^HIPT^N_3_N)]) discovered in the early 2000s, which was shown to catalyze N_2_ → NH_3_ conversion at room temperature^8–10^ (Fig. 1). This molybdenum-based catalyst operates through a series of proton-coupled electron transfer steps that activate the N_2_ moiety and yields NH_3_ according to eqn (1), where lutidinium (Lut) acts as proton donor and decamethylchromocene 
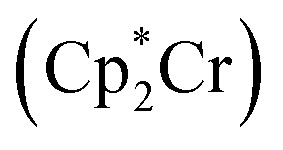
 acts as electron donor.1
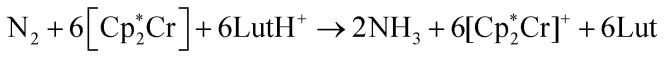


In the Schrock catalyst, molybdenum is in a trigonal bipyramidal configuration. However, the other examples also display molybdenum's ability to exist in stand alone or bridged octahedral configurations, which is a key aspect when designing new molybdenum based catalyst candidates. By understanding the mechanisms and efficiency of these complexes, one can obtain ideas and frameworks for future work on designing such catalysts. There have been many computational studies on the Schrock catalyst that provide crucial insights into the requirements of catalyzing the formation of NH_3_ (ref. 11–16) and these provide the foundation for further catalyst discovery studies.

Advances within computational chemistry, particularly generative models, have opened new frontiers in designing catalytic systems. Generative models mainly consist of machine learning (ML) based methods but can also include the more traditional methods like a Genetic Algorithm (GA). A GA can be seen as a conditional generative model, where the GA generates individuals that are targeted towards a certain fitness score. It can be used to combine existing molecules to screen toward a target property, or it can be used to combine existing molecules into unseen and novel structures through crossover. Generative models such as GAs have been shown efficient at screening chemical space for various chemical tasks^17–20^ and by combining a GA with ML or fast quantum method calculators, one can quickly explore chemical space for any desired property for a wide range of chemical challenges. Recent years have seen some highly promising work within GAs for catalyst discovery.^21–23^

In recent work we made efforts to optimize the Schrock catalyst by changing the HIPT ligand substituent with a variety of substituents suggested by a GA.^24^ In this work we extend this approach and now change the ligands connected to the core molybdenum atom. The GA evolves homoleptic molybdenum complexes in octahedral and trigonal bipyramidal geometries that catalyze nitrogen fixation *via* the Schrock cycle. Previous studies have highlighted the difficulty of the first protonation of the N_2_ moiety of the Schrock catalysts which was in agreement with our work on modified versions of the Schrock catalyst where the first protonation was found to be endergonic with a free reaction energy of ≈17 kcal mol^−1^.^11,24^ Modifying substituents on the ([(^HIPT^N_3_N)]) ligand did not in any cases result in a significant decrease of the first protonation which highlighted the difficulty of this step. As such, we attempt to improve this step with a GA. We start from the first reactant (Mo–N_2_) and use the first proton and first electron transfer steps as the fitness metric in the GA.

We use a modified GA approach based on the work of Seumer and Jensen^25^ combined with semi-empirical quantum methods to explore a vast array of possible ligands and 3D configurations of ligands through evaluation of potential monodentate ligands based on their ability to bond to molybdenum and their effectiveness in catalyzing the first charge transfer steps of the Schrock cycle. Ligands are continuously refined through natural selection and molecular crossover and in each evolution, a pre-screening process is performed to find optimal stereoisomers and coordinating atoms for candidate ligands and molybdenum combinations. Synthetic accessibility scores are used to guide the ligand search. We present the computational framework, the selection criteria for potential catalysts, and the subsequent computational validation of discovered catalysts. This approach not only contributes to the fundamental understanding of homogeneous nitrogen fixation catalysis but also sets a precedent for the application of computational tools in sustainable organometallic catalysis development in the future.

## 2. Methods

The objective for the GA is to find suitable ligands that combined with the Mo atom can form organometallic catalysts that effectively catalyze the first protonation (Mo–N_2_ → Mo–N_2_H^+^) and first reduction (Mo–N_2_H^+^ → Mo–N_2_H) reaction steps in the Schrock cycle. The main design considerations for such an approach are the number of coordinating ligands, the choice of coordinating atoms in the ligands and the stereoisomers for 3D catalyst structures.

The Schrock catalyst was used as a structural reference, where the quadridentate ([^HIPT^N_3_N]) ligand has three anionic coordinating sites in the equatorial position and one neutral coordinating site in the axial position. In the Schrock cycle, the Mo atom has formal oxidation states in the range (III–VI).^9^ The first intermediate of the cycle (Mo–N_2_) has a formal oxidation state of III. For our neutral catalysts to follow the same oxidation scheme, the catalysts should have exactly three anionic ligands. As such, the GA is designed to explore chemical space for catalysts with 3 anionic and either 1 or 2 neutral ligands. This means that Mo will have trigonal bipyramidal or octahedral coordination environments in the 1 and 2 neutral ligand cases respectively. Ligands of the same type (neutral or anionic) are constrained to be identical. The coordination environment of the molybdenum is set as an input parameter to the GA, and it will therefore only search for one configuration at a time.

### 2.1 Genetic algorithm

**Fig. 2:**
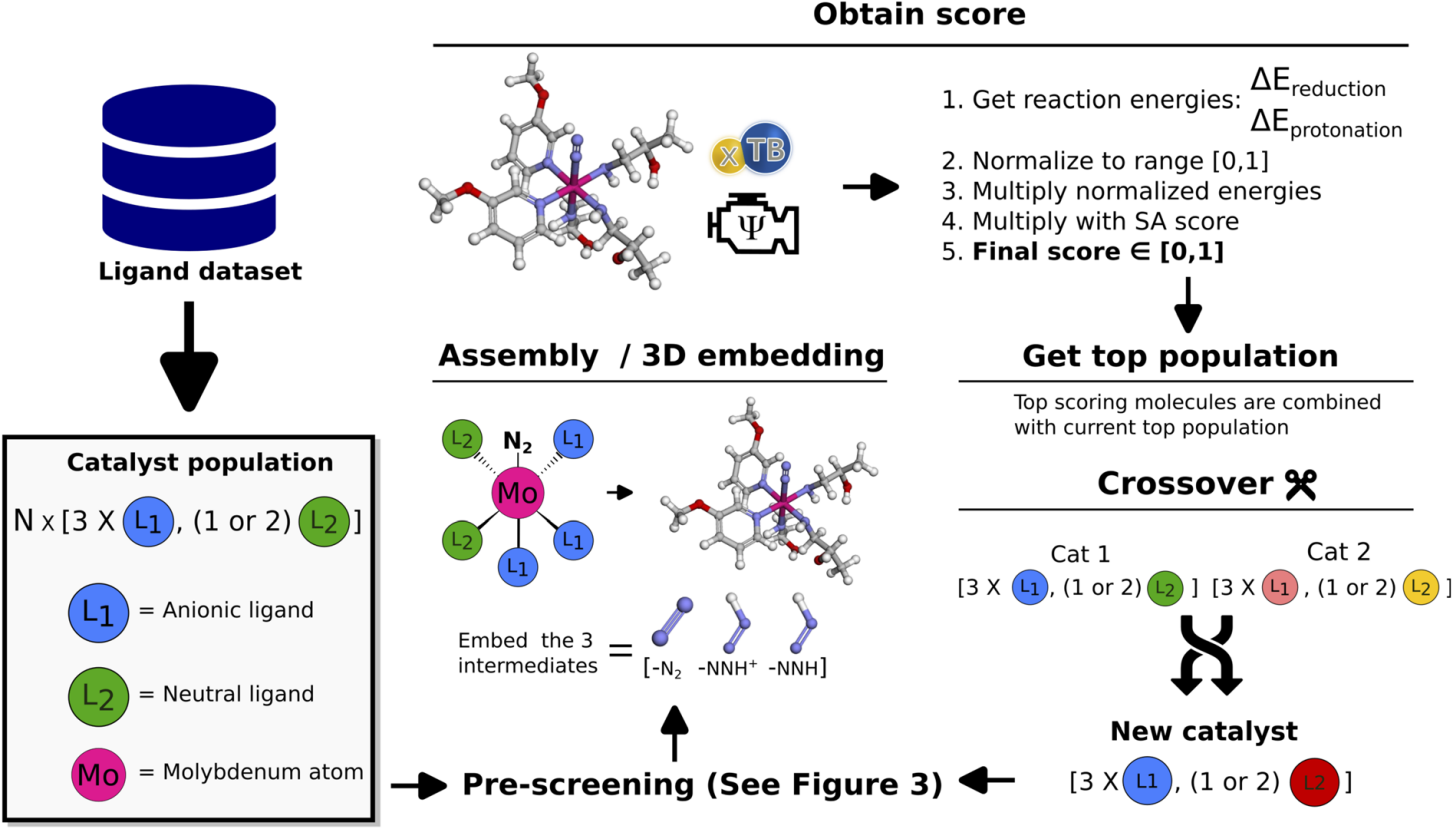
Schematic of GA workflow. Each catalyst is defined by a set of anionic and neutral ligands that are obtained from the Meyer *et al.*^26^ ligand dataset and passed to the algorithm. A detailed description of the crossover operations can be found in the ESI.†

The workflow of the GA is seen in Fig. 2. The dataset used as a starting point for GA runs consists of 91 ligands obtained from Meyer *et al.*^26^ including phosphines, pyridines and carbenes. From this dataset a pre-screening is performed followed by the score evaluation of catalyst structures which is used to determine the most promising ligands. The crossover and mutation operations follows the scheme presented by Jensen.^20^ Catalysts resulting from crossover are restricted to have between 2 and 100 heavy atoms and a maximum of 20 rotatable bonds. For a crossover operation between two catalysts (Fig. 2 crossover), one of the two catalysts is selected as the catalyst to be modified (Cat1 in Fig. 2). Then, a ligand is randomly selected from this catalyst and combined with the corresponding ligand type in the other catalyst. Each new catalyst after crossover will in this way have modified either the anionic or the neutral ligand, while keeping the other ligand unchanged. This prevents the crossover procedure from being too disruptive on the catalysts and enables steady evolution. For further details on the crossover operations see Fig. S10 in Section S4 in the ESI.†

#### 2.1.1 Pre-screening

**Fig. 3:**
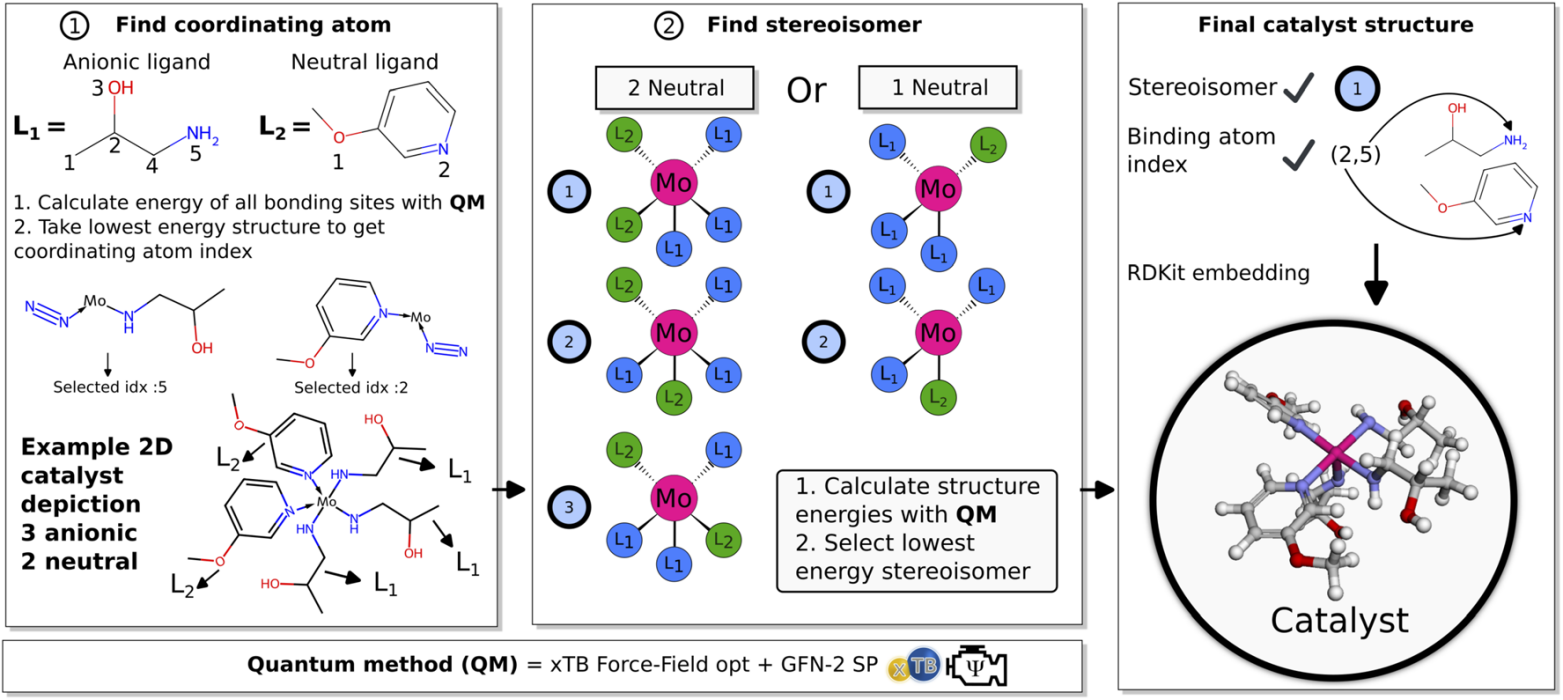
Illustration of the pre-screening process. (1) Coordinating atom check of proposed ligands for a catalyst. (2) Stereoisomer checks of the 3D catalyst structure. (3) Summary of selected parameters and final 3D catalyst structure.

Given a catalyst defined by a set of ligands, the coordinating atom and catalyst coordination environment is selected in a pre-screening process. An illustration of the pre-screening is shown in Fig. 3. The first step (1) is to find the coordinating atom in the ligands. Here, SMARTS pattern matching is used to find suitable coordinating atoms for either neutral or anionic ligands. For neutral ligands this entails carbenes, phosphines, amines or oxygens and for anionic ligands this is hydroxides, amines, halogens or SP3/SP2 carbons. Then, for all matching coordinating atom candidates the bonding energy between the ligand and a Mo–N_2_ core is calculated using an xTB-FF^27^ optimization followed by a GFN2-xTB^28^ single point (SP) calculation. The atom yielding the lowest ligand bonding energy is then selected as coordinating atom and the 2D graph of the catalyst can be defined. While this methodology gives chemically reasonable compounds and the energy difference between coordinating atoms identifies a single coordinating atom, the approach has not been systematically tested by comparison to experiment. We are not aware of any study like this in the literature and plan to pursue this important question in a future study.

Subsequently (2), we find the catalyst stereoisomer. Different permutations of the ligands in the 3D structure are created and afterward optimized with an xTB-FF optimization followed by a GFN2-SP calculation. The stereoisomer with the lowest energy is then chosen as the final catalyst structure which is embedded to create the final 3D intermediates that are evaluated in the scoring function.

#### 2.1.2 Scoring function

The scoring function in the GA takes a catalyst structure defined by the ligands, coordinating atoms and stereoisomers, calculates reaction energies for the first two reaction steps of the Schrock cycle based on 4 different conformers of the selected stereoisomer/coordination atom combination and yields two reaction energies. These are combined to give the final score for the GA to optimize. The reaction energies are obtained by structure optimizations of the embedded intermediates with GFN2-xTB.^28^ Reaction energies are based only on electronic energies to limit computation time in each generation. This approximation is supported by the fact that the reaction steps only involve proton and electron transfers, such that possible vibrational contributions are minor. The RDKit ETKDG method^29^ is used to go from SMILES representations to 3D structures. We apply an energy cutoff for the conformers where conformers with an energy higher than 20 kcal mol^−1^ relative to the lowest energy conformer are discarded. Additionally, a connectivity check is performed on the conformer geometry before and after optimization based on the overlap charge density from an extended Hückel calculation in RDKit with an overlap threshold of 0.15. If bonds are breaking during optimization, the conformer is discarded and can not be used for computing the score.

Previous work scored only on single reaction steps of the catalytic cycle.^24^ This proved to be somewhat effective. The GA did improve upon reaction energies used in the scoring function, at the cost of the introduction of significant barriers at other parts of the catalytic cycle. As such, in this work it was decided to include both types of charge-transfer in the scoring function by selecting the intermediates involved in the first charge (H^+^ + e^−^) transfers (N_2_, N_2_H^+^, N_2_H). Reaction energies are therefore obtained for the first protonation and first reduction of the N_2_ moiety. We denote the protonation reaction energy as *E*_p_ and the reduction energy as *E*_r_ and these reactions are seen in eqn (2) and (3). The shorthand form Mo–N_*x*_H_*y*_ is used as a label for the whole molybdenum plus ligand complex for a specific intermediate. The energies for proton and electron donors are also calculated with GFN2.2
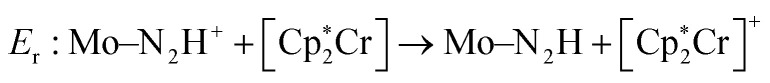

3 *E*_p_: Mo–N_2_ + LutH^+^ → Mo–N_2_H^+^ + Lut

The GA uses a single score value to determine the rank of scored molecules. Therefore, reaction energies are combined to a single value. This is done by first performing min–max scaling on the energies according to
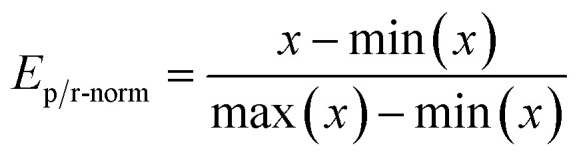


Here *x* is the reaction energy (units in kcal mol^−1^) and we set min(*x*) to 100 and max(*x*) to −100, which was based on empirical experience from the calculations on which ranges of reaction energies to expect. In this way, lower energies map to a higher value between 0 and 1 compared to higher energies. Molecules with reaction energies outside the normalization range are appropriately assigned to the extremum values of 0 and 1. Molecules with reaction energies less than −100 are assigned to the max score of 1 and molecules with energies higher than 100 were assigned to 0. The normalized energies are then multiplied together and finally scaled with an SA score as shown in eqn (4). The GA optimizing objective is to maximize the number resulting from this eqn (4) and how the SA_score_ is defined is discussed in the following.

4 Score = *E*_p-norm_ × *E*_r-norm_ × SA_score_

#### 2.1.3 Synthetic accessibility

To steer the GA towards synthetically accessible ligands, the Gaussian score modifier suggested by Gao and Coley^30^ is used together with the synthetic accessibility scoring function developed by Ertl and Schuffenhauer.^31^ Each catalyst consists of 3 anionic and either 1 or 2 neutral ligands, which means that each ligands will have different SA scores. Anionic ligands are neutralized before calculating the SA score and the SA is evaluated in two steps. First as the average of the individual SA score for all ligands. Secondly, an additional Gaussian modifier was applied to the average SA score. This modifier decreases the impact of the SA on the final score by squeezing the upper range of SA scores together. The idea was to diminish the impact of the SA score for catalysts that already had average SA scores above 0.5. This was done to prevent ligands with non-ideal SA scores at first, from being quickly neglected without any chance of improving through crossover. See Fig. S7 in the ESI† for further details.

**Fig. 4:**
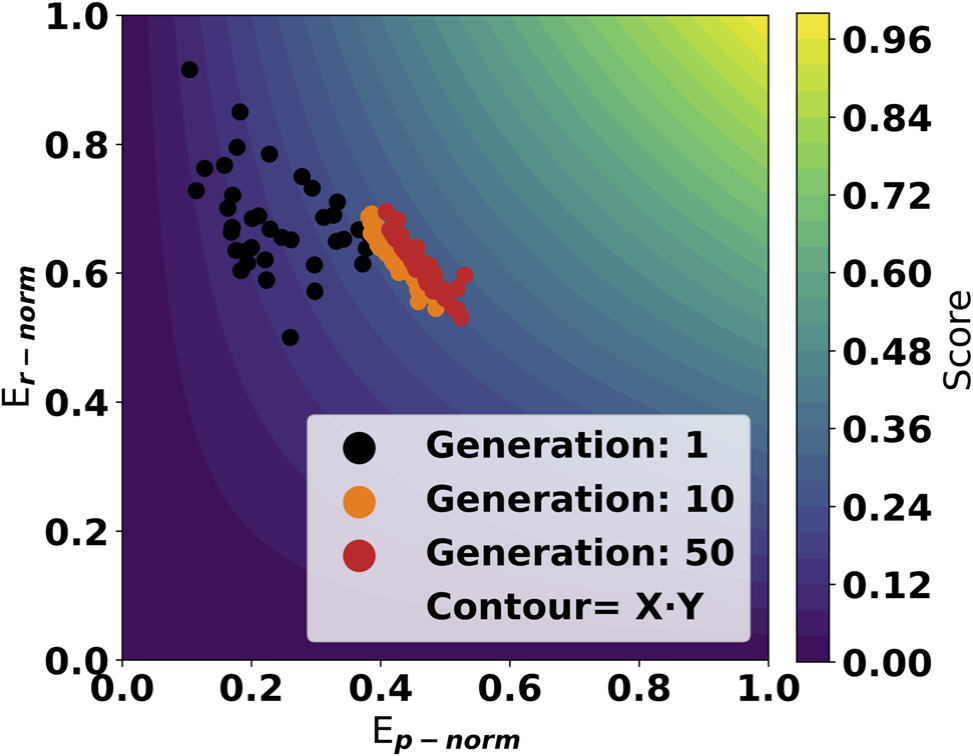
Normalized reaction energy scores for a GA run with a populations size of 50 and run for 50 generations. Each point in the figure correspond to the score from one molecule in a generation. The contour surface illustrates the total score at all points. The SA_score_ modifier is applied to the *x*-axis values.

A trial run using the scoring objective in eqn (4) is displayed in Fig. 4. From the first generations, the catalysts are scattered with high scores along the *y*-axis. As evolution progresses, the population moves towards a front of high scoring molecules in both dimensions. This highlights the effectiveness of using this approach as the GA can optimize in both dimensions on the GFN2 energy surface.

### 2.2 Validation of a selection of final populations

Thirty GA runs are performed with 1 neutral ligand configurations and sixteen GA runs with the 2 neutral ligands configurations. We take the final population of all of these GA runs and obtain 1332 unique catalysts for the 1 neutral ligand and 747 unique catalysts for the 2 neutral ligand configurations. As a first step of validating these catalysts, a thorough conformer search is performed for each catalyst. This is in essence re-doing the scoring for all catalysts with a significantly higher number of conformers. The SA score is not considered at this stage as we are no longer evolving ligands.

#### 2.2.1 Conformer search

For each catalyst intermediate we perform the ETKDG embedding with n_confs = 500 and a RMSD pruning threshold of 1. This is done to ensure a diverse conformer pool and in general results in approximately 50–300 conformers for each catalyst intermediate. Additionally, the stereoisomer step of the pre-screening (Fig. 3) is redone for each molecule with the increased number of conformers to ensure that the optimal stereoisomer is found. The coordinating atom for each ligand is kept the same. Additional results from the conformer search is shown in the ESI.†

#### 2.2.2 DFT validation

Catalysts that fail the conformer search for one or all of the three intermediates, are discarded. For the remaining catalysts we use the assembled TMC graph to get Morgan fingerprints with a radius of 3 and use this fingerprint to calculate Tanimoto scores. For all pairs of complexes that have similarity above 0.6 we randomly discard one of the complexes in the pair. This is done to keep the diversity of the selection high and left us with a pool of 387 catalysts with 1 neutral ligand and 235 catalysts with 2 neutral ligands. Subsequently, DFT single points are performed on the top 10 lowest energy conformers for each intermediate in each catalyst. The conformers yielding the lowest DFT energies are then used to re-compute the catalyst score (without SA modification). The DFT single points are performed at the def2-TZVP/PBE level of theory and we use the CPCM^32^ solvent model with *ε* = 1.844. The DFT methods applied are identical to the work done by Thimm *et al.*^11^ and all DFT related details are explained in Section S5.2 in the ESI.†

These DFT SP scores are used to discard molecules with low scores as it was not computationally feasible to do DFT verification on all molecules. A cutoff of 10 kcal mol^−1^ is used for both protonation and reaction energies. This corresponds to a normalized value of 0.45. For the remaining molecules, the lowest DFT SP energy conformers for each of the 3 scoring intermediates are optimized with def2-TZVP/PBE yielding electronic energies for DFT relaxed structures that are used to compute final DFT based reaction energies. Here we once again apply the 10 kcal mol^−1^ reaction energy cutoff to limit the number of catalysts in the computation pool. This left a population of 96 catalysts with 1 neutral ligand and 70 catalysts with 2 neutral ligands. For these catalysts we attempt to calculate the full Schrock cycle reaction profile. We use the retrosynthesis tool Manifold^33^ on these final catalyst populations to predict the minimum number of synthetic steps required to synthesize each ligand from commercially available building blocks. All catalyst ligands were found to have 3 or fewer synthetic steps.

#### 2.2.3 xTB and DFT correlation

**Fig. 5:**
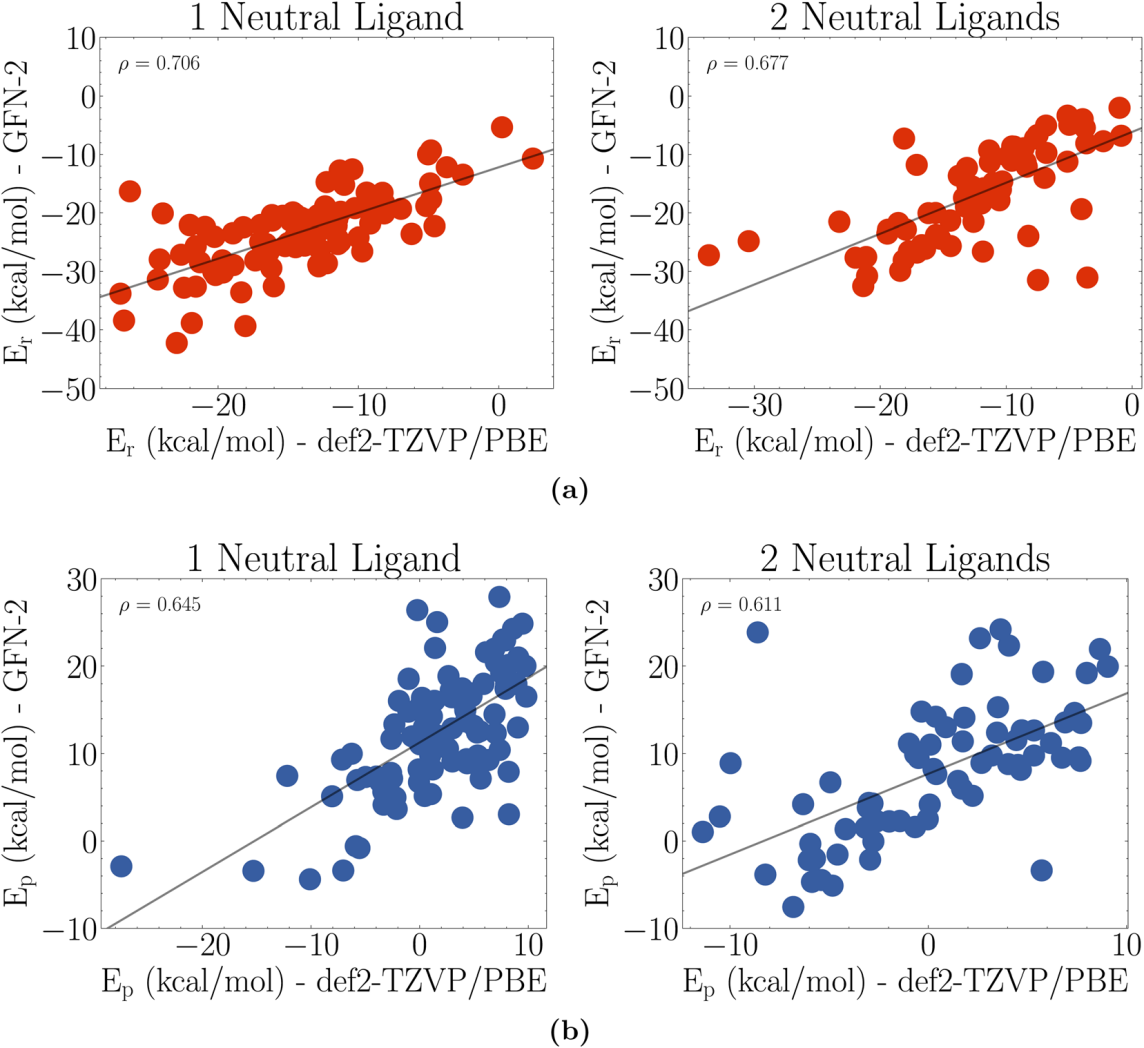
Comparison of GFN2-xTB optimized reaction energies against def2-TZVP/PBE optimized reaction energies. (a) Reduction energies and (b) protonation energies. Here *ρ* is the Pearson correlation of the data.

The protonation and reduction reaction energies from GFN2-xTB and def2-TZVP/PBE calculations on the final population of [96,70] molecules are compared in Fig. 5. There is a clear Pearson correlation observed with Pearson correlation coefficients above 0.6 for all cases. It is not an ideal correlation, but it is sufficient to demonstrate the capability of the GA to rapidly search through thousands of catalysts with methods that require only a fraction of the computational cost compared to higher level quantum methods.

### 2.3 Calculating full reaction profiles

After obtaining the final pool of catalysts ([96,70]) we try to calculate full catalytic cycles for all catalysts. We do not consider the pathway involving the six coordinated Mo–NH_3_–N_2_ intermediate, but rather the Mo intermediate. The optimized PBE Mo–N_2_ geometry for each catalyst is used as a reference to create the first intermediate in the Schrock cycle. Each catalytic cycle is optimized sequentially, which means that when an intermediate is done optimizing the next optimization use this optimized intermediate as the starting structure. This simulates a better reaction flow compared to using the same starting structure for all 15 intermediates. The N_*x*_H_*x*_ moieties are then embedded on to this starting structure with a GFN2 pre optimization of the N_*x*_H_*x*_ atoms, to ensure a reasonable starting structure for DFT. All other atoms are fixed. If an PBE optimization fails for an intermediate, the converged Mo–N_2_ geometry is used as starting structure, If that also fails the catalyst is discarded as all 15 intermediates are needed to be able to get full catalytic cycles. The final intermediate energies are obtained with def2-TZVP/B3LYP SP on the PBE optimized structures. Due to computational limitations, GFN2-xTB is used to compute vibrational corrections.

## 3. Results

### 3.1 Final population characterization

**Fig. 6:**
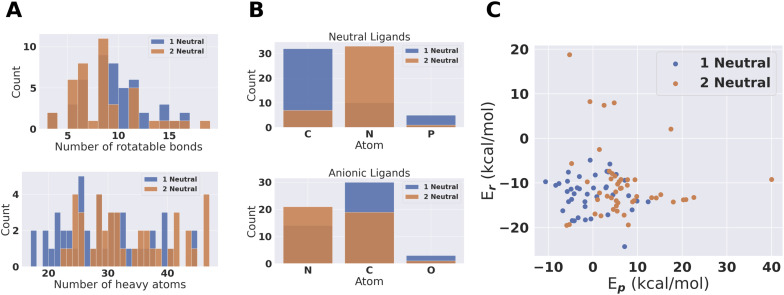
Population characteristics of the 41 + 47 catalysts where full catalytic cycles were obtained. (A) Rotatable bonds and number of heavy atoms in the catalysts. (B) Coordinating atoms for the two types of ligands. (C) PBE optimized energies for the catalysts.

From the pool of (96 + 70) catalysts we obtain 47 fully PBE optimized catalytic cycles for 1 neutral and 41 for 2 neutral ligand configurations. This pool of catalysts was characterized based on number of atoms, number of rotatable bonds and coordination atoms. The result is shown in Fig. 6. The largest catalyst has approximately 50 heavy atoms, even though catalysts with up to 100 heavy atoms where allowed. The larger catalysts could therefore not obtain sufficiently large scores to be accepted during conformer search and DFT SP screening. Amines are clearly preferred coordination environments for neutral ligands for the 2 neutral ligand configuration. On the other hand, the 1 neutral ligand configuration prefers carbenes. We also note that phosphines are present in the final compounds. Phosphines and carbenes usually get low SA scores. The use of an additional Gaussian modifier SA procedure allowed such ligands to evolve if the reaction energy improvement was sufficient to overcome the decrease in score due to the SA modifier.

For the anionic ligands, the vast majority of coordinating atoms are carbons or nitrogens for both the 1 and 2 neutral ligands configurations. Anionic carbon atoms are not the most commonly found coordinating atoms for TMC ligands. For monodentate ligands with carbon coordinating atoms in the tmQMg-L, the carbene coordinating environments are dominating.^34^ However, we looked at the neutral monodentates in the tmQMg-L and found that there are 327 ligands where the coordinating atom is an anionic carbon which supports the use of these anionic carbon ligands in our approach. How this analysis was done is explained in Section S7 in the ESI.† Subsequently we will look further into the 3D structures and reaction profiles of the catalysts from Fig. 6. All catalyst reaction profiles are compared to the Schrock catalyst reaction profiles calculated at the same level of theory (S5.1†).

### 3.2 Catalysts with 1 neutral ligand

**Fig. 7:**
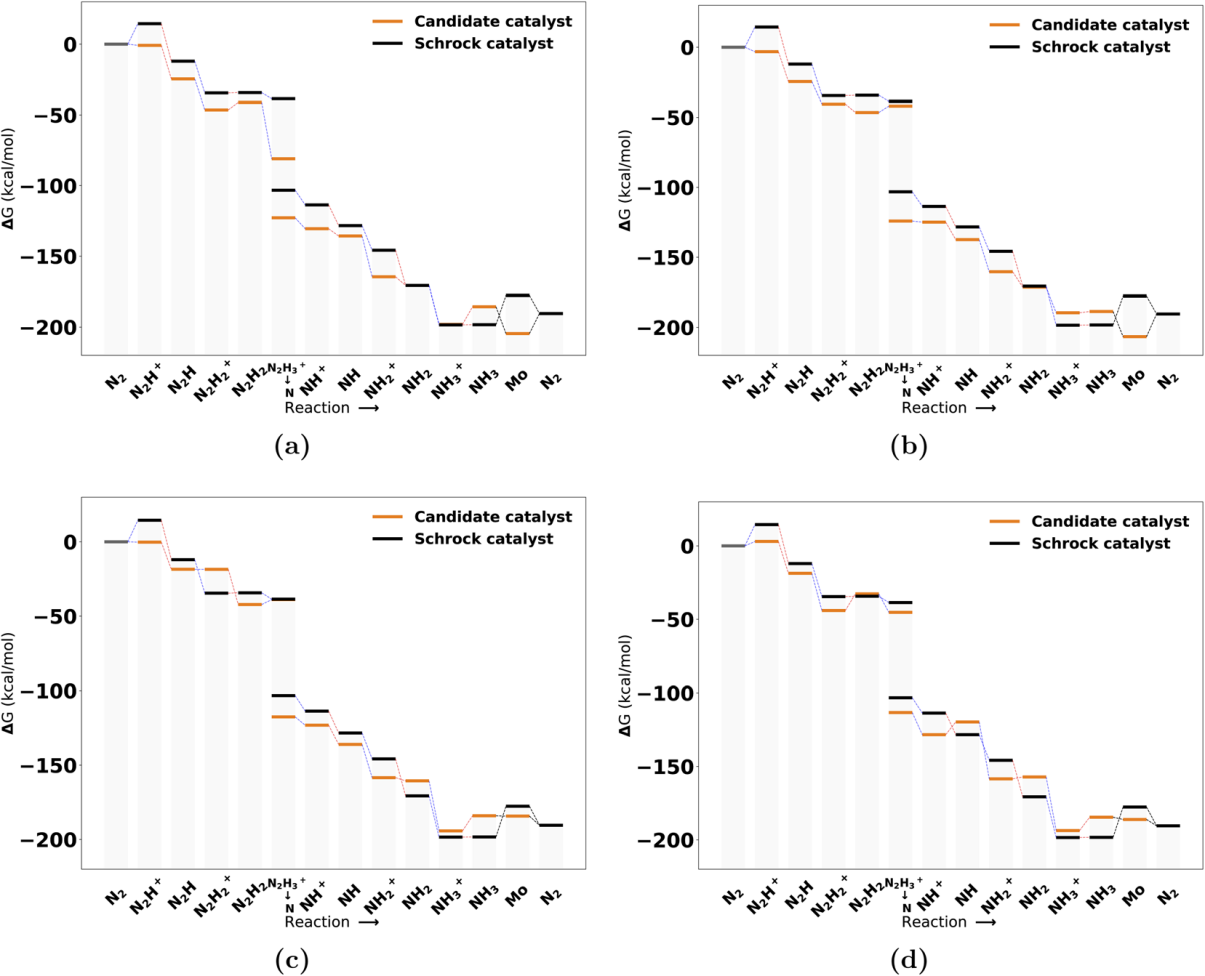
Reaction profile for the 4 top scoring catalysts with 1 neutral ligand at the B3LYP level from highest (a) to lowest (d). All energies are obtained from PBE optimized structures with subsequent B3LYP single points. Blue lines connecting energy levels indicate proton transfer and red lines indicate electron transfer. The short hand labels on the *x*-axis refer to the state of the N_*x*_H_*y*_ moiety on each intermediate.

The catalysts with 1 neutral and 3 anionic ligands follow the same coordination environment as the Schrock catalyst. The difference being that the Schrock catalyst has a quadradentate ligand, whereas we apply 4 monodentate ligands. For the pool of catalysts obtained with one neutral ligand, 47 full cycles were obtained. A final energy refinement is performed on these 47 catalysts with def-TZVP/B3LYP SP calculations and we calculate vibrational corrections with GFN2-xTB. The SA score is not considered here as these catalysts have already made it through the SA screening and have 3 or fewer synthetic steps from Manifold. The free energy profiles of the four top scoring examples are shown in Fig. 7. The first thing to notice is the clear effect from the scoring function as the first protonation and reduction steps are exergonic and nearly exergonic for 7 d, whereas for the Schrock catalyst the first protonation step is endergonic. Other parts of the cycle are fairly similar to the reference. The release of NH_3_ to form the Mo intermediate is also observed to be more accessible than the reference, at the cost of an increased barrier to reduction (NH_3_^+^ → NH_3_). A full overview of the top 10 best scoring catalysts is found in Fig. S13 in the ESI.†

Inspection of the catalytic cycles for converged 1 neutral ligand structures revealed that all catalyst cycles had one or more intermediates that had converged to configurations where the catalysts could no longer be recognized as trigonal bipyramidal. Three examples of this is shown in Fig. S11 in the ESI.† The Mo–N intermediate in particular proved to be a difficult case to handle for the catalysts. This highlights the complexity of designing organometallic catalysts for longer reaction mechanisms. The rearrangement of the structure was not observed for the catalysts with 2 neutral ligands. The 1 neutral ligand configurations were therefore not pursued further and the remaining analysis will focus on the octahedral configurations of the catalysts.

### 3.3 Catalysts with 2 neutral ligands

**Fig. 8:**
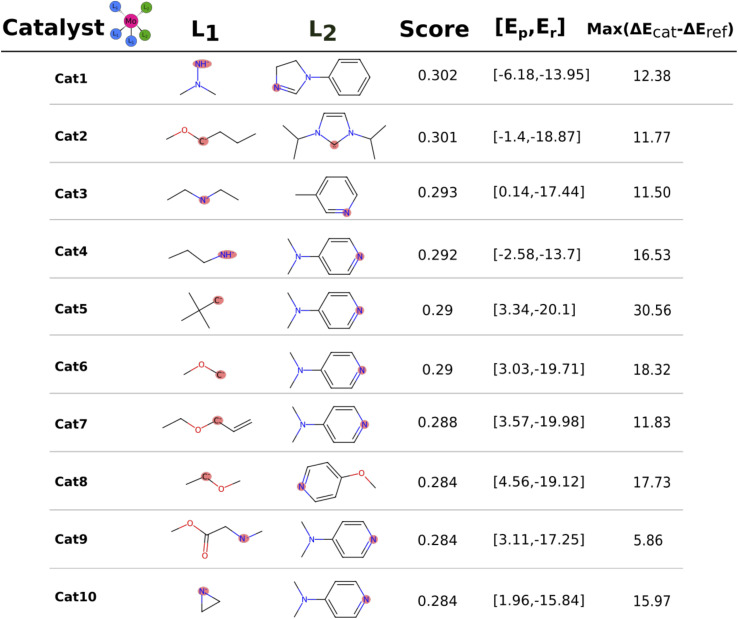
Top 10 molecules after the final B3LYP energy refinement. Every catalyst is in a octahedral coordination geometry with 3 × L_1_ and 2 × L_2_. The score column contains the score based on the multiplication of normalized electronic reaction energies. The second to last column is the reaction energies (kcal mol^−1^) and the last column denotes the largest increase in reaction energy relative to the reference Schrock catalyst across the catalytic cycle.

The catalysts with 2 neutral and 3 anionic ligands have one higher coordination number for the central molybdenum as compared to the Schrock catalyst. We follow the same energy refinement procedure as for the 1 neutral ligand catalysts above where we here attempt to do def-TZVP/B3LYP SP refinements on the 41 PBE reaction profiles and succeed for 36. An overview of the top scoring catalysts without vibrational corrections are shown in Fig. 8. A clear trend is seen for all ten catalysts, the first protonation of the N_2_ moiety is either equal or lower in energy compared to the Schrock catalyst, while the reduction energies are all negative. Cat5 has the largest increase in reaction energy which occurs at the NH_3_^+^ → NH_3_ reduction step. Furthermore, there is a pattern in ligand types where for the anionic ligands, smaller chains are favored for the top 10 catalysts. For neutral ligands, we observe several identical ligands. For example, Cat{4,5,6,7,9,10} have the same pyridine like ligand. A single carbene ligand is observed for Cat2. To keep the discussion limited, the top 3 catalysts are selected for further inspection of the energy reaction profiles where we add vibrational corrections. All structures and calculation data for the remaining energy profiles are available in the ESI.†

#### 3.3.1 Cat1

**Fig. 9:**
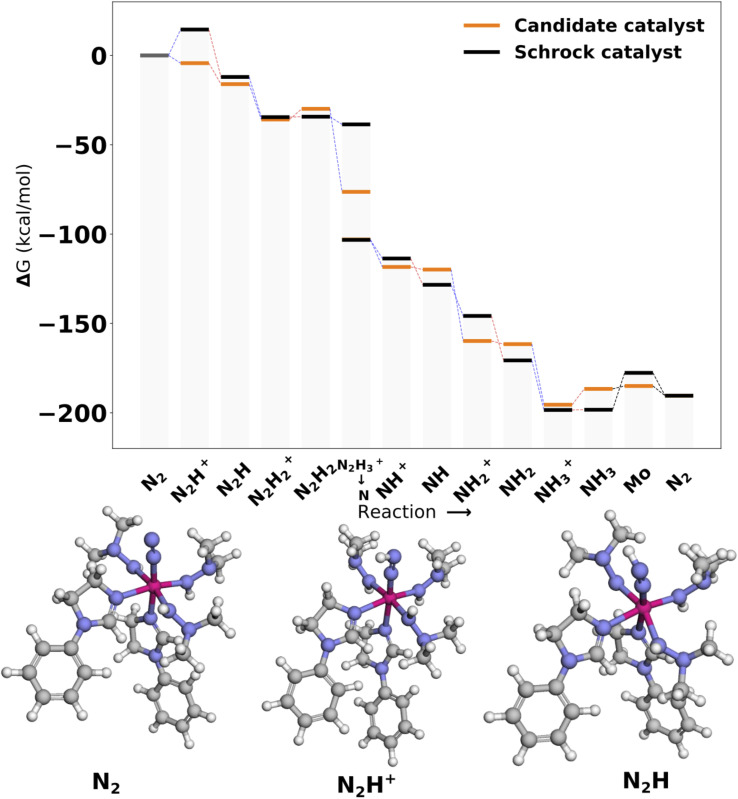
Reaction profile for Cat1. All energies are obtained from PBE optimized structures with subsequent B3LYP single points. The optimized 3D structures for the first intermediates are shown at the bottom. Blue lines connecting energy levels indicate proton transfer and red lines indicate electron transfer.

Fig. 9 shows the reaction profile for the top scoring catalyst Cat1. We observe that the first protonation and reduction scoring steps are exergonic (−4.33 and −16.06 kcal mol^−1^), in accordance with the scoring function. Overall the energy profile appears fairly similar to the reference. A slight reduction barrier is introduced for N_2_H_2_^+^ → N_2_H_2_ and a larger barrier is introduced for NH_3_^+^ → NH_3_. Lastly, we should note the N_2_H_3_^+^ state is lowered significantly. This is due to the fact that the proton on the N_2_H_3_^+^ moiety moves to an equatorial nitrogen during optimization. This optimized 3D structure can be found in Fig. S12 in the ESI.†

#### 3.3.2 Cat2

**Fig. 10:**
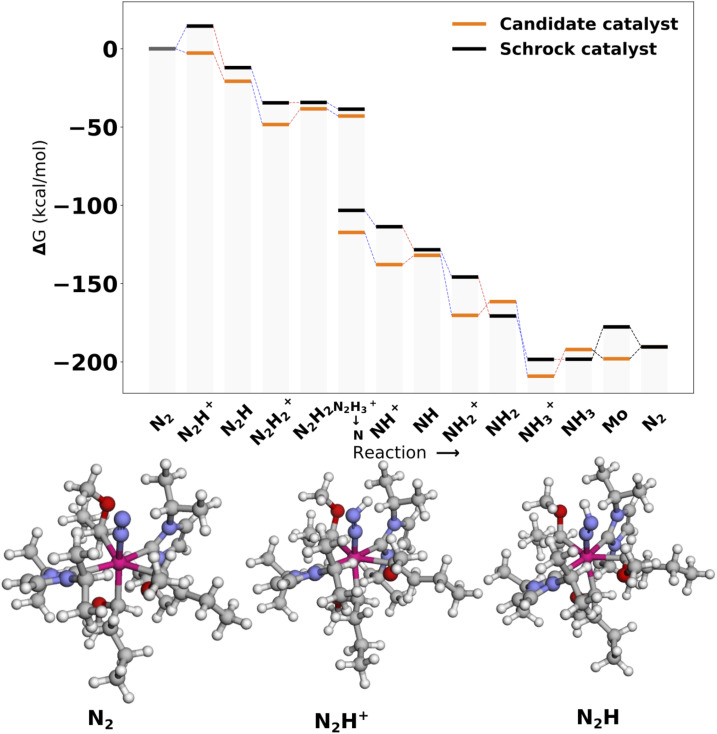
Reaction profile for Cat2. All energies are obtained from PBE optimized structures with subsequent B3LYP single points. Blue lines connecting energy levels indicate proton transfer and red lines indicate electron transfer.

Fig. 10 shows the reaction profile for Cat2, the second best scoring catalyst. The protonation energy is −2.75 kcal mol^−1^ and is once again lower than the reference. Furthermore, protonations are heavily favored across the whole profile. This comes at the cost of less favorable reductions. In this case, the inclusion of the first reduction in the scoring function did not seem to transfer effectively to other reduction steps.

#### 3.3.3 Cat3

**Fig. 11:**
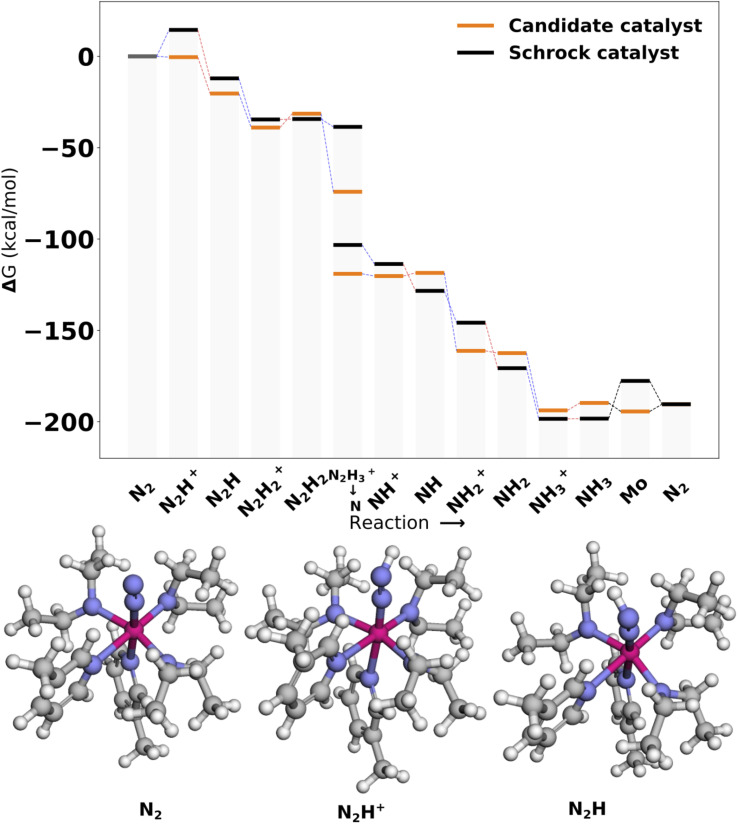
Reaction profile for Cat3. All energies are obtained from PBE optimized structures with subsequent B3LYP single points. Blue lines connecting energy levels indicate proton transfer and red lines indicate electron transfer.

Fig. 11 shows the reaction profile for Cat3, the third best scoring catalyst. We note that the scoring step is clearly improved with a protonation energy of −0.43 kcal mol^−1^. Similarly to Cat1, we see barriers introduced for the N_2_H_2_^+^ → N_2_H_2_ and NH_3_^+^ → NH_3_ reactions. Additionally, the energy profile is fairly similar to the reference and does not contain the same reduction barriers introduced for Cat2. We observe that the N_2_H_3_^+^ intermediate is significantly lowered in energy, which is also due to the migration of the proton to an equatorial nitrogen. The 3D structure of this intermediate can be found in Fig. S12 in the ESI.†

#### 3.3.4 Ligand contributions to protonation energy

To gain insight into how the negative protonation energy is achieved we systematically replace negative and neutral ligands by NH_2_^−^ and NH_3_, respectively, keeping the metal–ligand distance unchanged. We start replacing each of the 5 ligands individually and then we recompute the electronic protonation energy to determine the effect of removing each ligand. Then, we order the ligands from largest positive increase to lowest positive increase in energy, and use this order to subsequently remove one by one ligand and recompute electronic protonation energy.

**Fig. 12:**
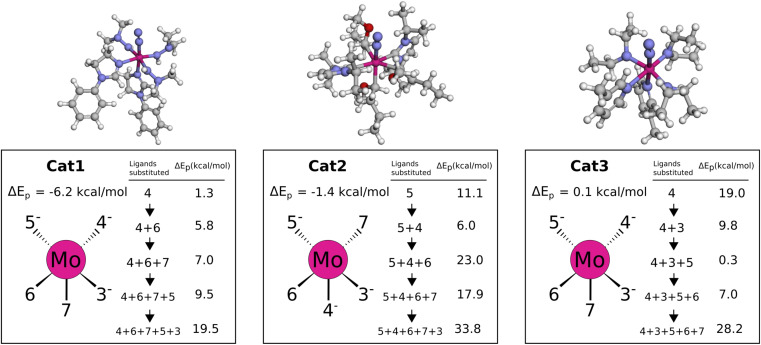
Protonation energies for various ligand substitutions with NH_2_^−^ and NH_3_ for Cat{1,2,3}. Energies are based on B3LYP single points of the def2-TZVP/PBE optimized Mo–N_2_ and Mo–N_2_H^+^ intermediates. The orientation of the catalyst sketches in each box matches the 3D structure shown at the top.

In Cat1 the three negative hydrazine ligands (3, 4, and 5) are all equatorial as shown in Fig. 12, whereas the neutral ligands are 2,4-dihydroimidazoles. The ligand that makes the single largest contribution to the protonation energy is the negative ligand (ligand 4), trans to the equatorial neutral ligand (ligand 6), followed by ligands 6, 7 (the axial ligand), 5, and 3. Changing ligand 4 to an NH_2_^−^ increases the protonation energy from −6.2 to +1.3 kcal mol^−1^. Changing both ligands 4 and 6 increases the protonation energy further to +5.8 kcal mol^−1^, and the protonation energy remains positive for all other substitutions as shown in Fig. 12. While this analysis points towards ligand 4 as the main reason for the exergonic protonation energy, it should be noted that the ligand-effects can be significantly non-additive as shown in Fig. S15.†

In Cat2 the negative and neutral ligands are carbanions and carbenes, respectively. Contrary to Cat1, the axial ligand (ligand 4) is now negative and the equatorial negative (ligands 3 and 5) and neutral ligands (ligands 6 and 7) are both trans. The ligand with the single largest effect on the protonation is one of the equatorial negative ligands (ligand 5 in Fig. 12). Exchanging this ligand with NH_2_^−^ increases the protonation energy from −1.4 to 11.1 kcal mol^−1^. All subsequent substitutions yield structures with positive protonation energies.

In Cat3 the negative and neutral ligands are secondary aliphatic amines and pyridines, respectively, where the ligands are arranged as in Cat1 (Fig. 12). As with Cat1, the ligand that makes the single largest contribution is negative ligand 4, which increases the protonation energy from 0.1 to 19.0 kcal mol^−1^ when replaced with NH_2_^−^. Interestingly the following three substitutions all lower the protonation energy.

While the fully substituted structures are chemically identical they have different bond lengths and angles, which account for the differences in their protonation energies ([19.5, 33.8, 28.2]). This highlights that the discovered ligands also affect the protonation energy by inducing structural changes.

### 3.4 Volcano plot

**Fig. 13:**
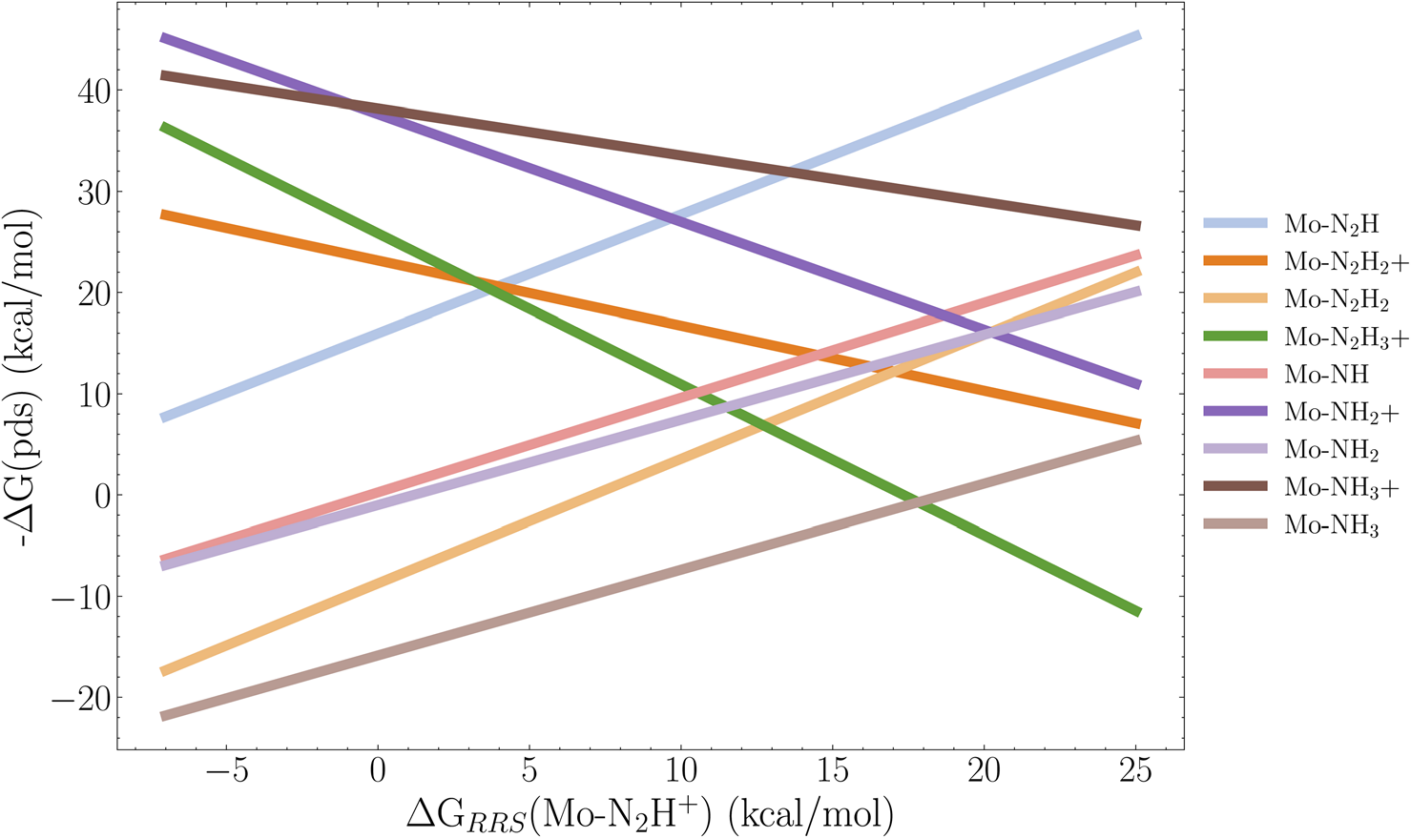
Volcano plot based on the linear scaling relations for 9 reactions based on the 36 B3LYP SP energy profiles of the catalysts with 2 neutral ligands. The *x*-axis descriptor is the first protonation energy in the cycle relative to the resting state, and each legend label refers to the resulting intermediate of a reaction. For example Mo–N_2_H = Mo–N_2_ → Mo–N_2_H. The scaling relations used to construct this figure are found the ESI (Fig. S14†). Pds is the potential determining step.

It is clear from Fig. 10–12 that lowering the protonation energy affects the energies of several other steps in the catalytic cycle. To investigate this further we collect the B3LYP SP reaction energies for all catalysts with 2 neutral ligands (36 catalysts). Due to the structural distortions of the 1 neutral ligand catalysts, these were not considered for this analysis. Following the approach from Busch *et al.*^35^ we then construct the scaling relations between all reactions and the first protonation reaction relative to the resting state which is the Mo–N_2_ intermediate. The individual scaling relations and their *R*^2^ value can be found in Fig. S14 in the ESI.† We set a cutoff of 0.2 for the coefficient of determination. Scaling relations with *R*^2^ below this value are not considered to be relevant for the first protonation energy. The resulting volcano plot is shown in Fig. 13.

We observe that the limiting step at lower protonation energies is the reduction reaction Mo–NH_3_^+^ → Mo–NH_3_, followed closely by the Mo–N_2_H_2_^+^ → Mo–N_2_H_2_ reduction, as the energy of these steps tends to increase when the protonation energy is lowered. The top of the volcano appears at around 17 kcal mol^−1^. Here the protonations Mo–N_2_H_2_ → Mo–N_2_H_3_^+^ and Mo–N → Mo–NH^+^ start to become the limiting factors. It appears that to have an exergonic first protonation we need to also consider the Mo–NH_3_^+^ → Mo–NH_3_ and Mo–N_2_H_2_^+^ → Mo–N_2_H_2_ reactions in the GA, since all the cases where the protonation energy is negative, the reduction energy is positive. However, we note that even though there appears to be introduced barriers for the Mo–NH_3_^+^ → Mo–NH_3_ reaction, the overall energy profile of the last 3 reaction steps of Schrock cycle (Mo–NH_3_^+^ → Mo–N_2_) is improved for all three top scoring catalysts (Cat1,2,3) in Fig. 9–11 as the release of NH_3_ is more favourable. A final note is that it would be relevant to explore GA objectives where the target protonation value is a range between 0 and −5 kcal mol^−1^ instead of a pure minimization, in order to find a compromise between a negative protonation energy and a low barrier Mo–NH_3_^+^ → Mo–NH_3_ reaction. Given the complexity of the task, the GA performed extremely well at lowering the protonation energy, while preventing significant barriers for the remaining 14 steps in the cycle.

## 4. Conclusion and outlook

This study applies a Genetic Algorithm(GA) to discover possible molybdenum based catalysts for nitrogen fixation with a fast semi-empirical quantum method (GFN2-xTB). The catalysts are restricted to follow two types of ligand coordination, one with 3 anionic ligands and 1 neutral ligand and the other with 3 anionic ligands and 2 neutral ligands. We use a dataset of 91 experimentally verified ligands as the starting population for the GA and use a pre-screening procedure to assign coordinating atoms to candidate ligands and assign the ligand permutations in trigonal bipyramidal and octahedral configurations. The optimizing objective is the first protonation and reduction steps of the Schrock cycle and we find multiple candidates that appears at B3LYP level of theory to inherently improve upon these two first charge transfer steps while keeping the effect on other reaction barriers minimal.

We find complexes for which the protonation energy is negative for both four- and five-ligand complexes, but for the former the structures deviate significantly from the ideal trigonal bipyramidal geometry. Through analysis of three of the 5-ligand complexes we identify a (negative) ligand which makes the single largest contribution, but the analysis is complicated by large non-additive effects. We do show that in addition to the metal ligand interactions, the ligand–ligand interactions also contribute to the low protonation energies by small structural distortions.

By analysis of 36 complexes with 2 neutral ligands we construct a volcano plot and observe how the energy of the initial protonation step is strongly tied to the Mo–NH_3_^+^ → Mo–NH_3_ and Mo–N_2_H_2_^+^ → Mo–N_2_H_2_ reactions, which makes the discovery of efficient catalysts for this reaction difficult.

We only consider three intermediates in the scoring function and future work should be expanded to include even more intermediates if computationally feasible. In particular the reduction steps Mo–NH_3_^+^ → Mo–NH_3_ or Mo–N_2_H_2_^+^ → Mo–N_2_H_2_ would be of high interest. Additionally, expanding the scope of the GA to include other transition metal atoms is a relevant direction to explore.

## Data availability

The DFT data is available at https://sid.erda.dk/sharelink/ExOVgOnP8P and the code for the genetic algorithm is available at https://github.com/jensengroup/genetic_algorithm_for_nitrogen_fixation.

## Author contributions

M. S. performed all the data curation, performed DFT calculations and analyzed the results. M. S. wrote the manuscript in collaboration with J. J. J. J. supervised the project and M. S. and J. S wrote the code for the Genetic Algorithm in collaboration.

## Conflicts of interest

There are no conflicts to declare.

## Supplementary Material

SC-015-D4SC02227K-s001
