# Supplementary material for: Discovery of molybdenum based nitrogen fixation catalysts with genetic algorithms
Source: Chem Sci. 2024 Jun 7;15(27):10638–50. doi: 10.1039/d4sc02227k (PMC11234868; doi:10.1039/d4sc02227k)
Supplement: SC-015-D4SC02227K-s001 [file SC-015-D4SC02227K-s001.pdf]

Electronic Supplementary Material (ESI) for Chemical Science.
This journal is © The Royal Society of Chemistry 2024

Supporting Information

S1 Conformer search and DFT verification

Figures S1 show the results from the conformer search. One can clearly see how the pool of molecules is heavily decreased by the conformer search. Ideally the quality of the GA output should be high enough that most or all of them should be viable molecules after the search. However, only 4 conformers where used during GA runs. As such, the stereoisomer checks where done with few conformers and it is possible that increasing the number of conformers tried for each ligand orientation would lead to new configurations and then these configurations would not be stable when performing a GFN2 optimization. Nevertheless, the conformer search ensured that the molecules that remained where likely to have a more accurate representation of the score and to have more reasonable geometries.

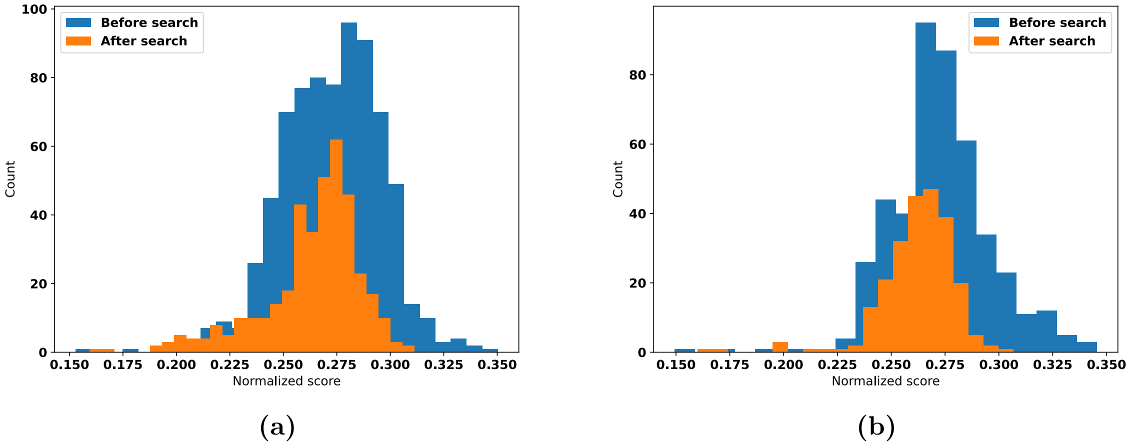

Figure S1: Comparison of scores and population size before and after conformer search for **(a)** 1 neutral ligand and **(b)** 2 neutral ligand configurations. The score is based on the energy of the lowest energy conformer. The decrease in number of molecules after the search is due to the fact that some molecules did not obtain any valid conformers in the conformer search.

After the conformer search the 10 lowest energy conformers where passed to def2-TZVP/PBE single point calculations to obtain new lowest energy conformers at the DFT level. Then the molecules where filtered based on these new scores as described in the main text. An example of how this SP refinement helped select better structure is shown in Figure S2. The final verification step was the def2-TZVP/PBE optimization of the new lowest energy conformers. The resulting reaction and protonation energy distributions for the 1 neutral and 2 neutral ligand configurations are shown in Figure S3 and Figure S4. At this level where conformers are def2-TZVP/PBE optimized there is a large portion of molecules with positive protonation energies (Fig. S3 and Fig. S4). This is where we applied the second filter to obtain the final populations (90+70) for which we attempt to compute full catalytic cycles. A random selection of 40 of these catalysts are seen in Figure S6 for the 1 neutral ligand catalysts and in Figure S6 for the 2 neutral ligand catalysts.

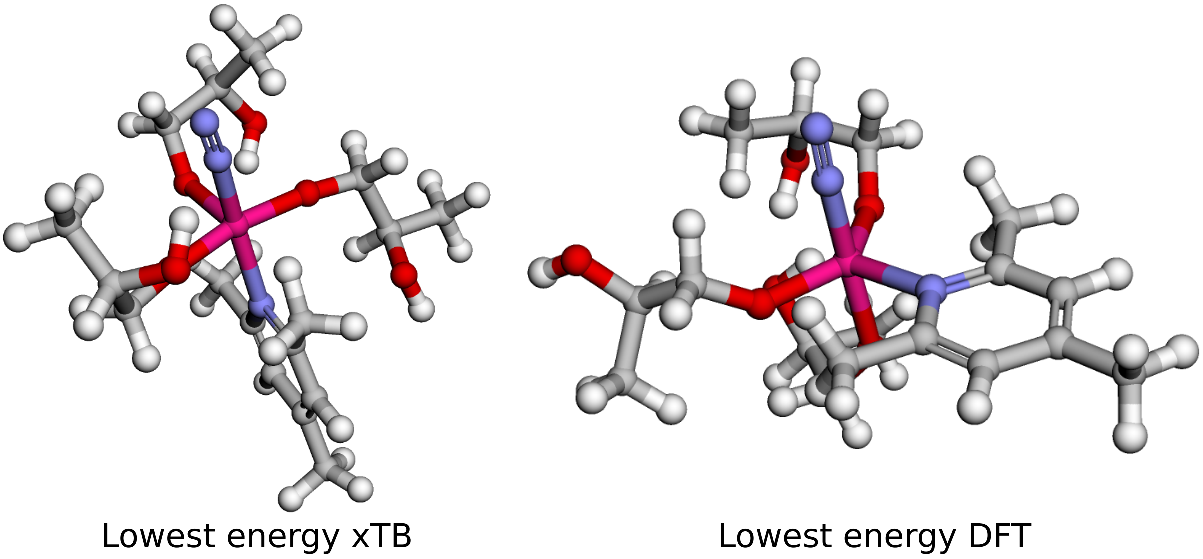

Figure S2: Example of the disparity between DFT and xTB lowest energy structures. It appears that the lowest energy xTB has been embedded with a different starting structure. This is not the case, the optimization of the xTB structure has moved the neutral ligand into an axial position in stead of equatorial as seen on the right.

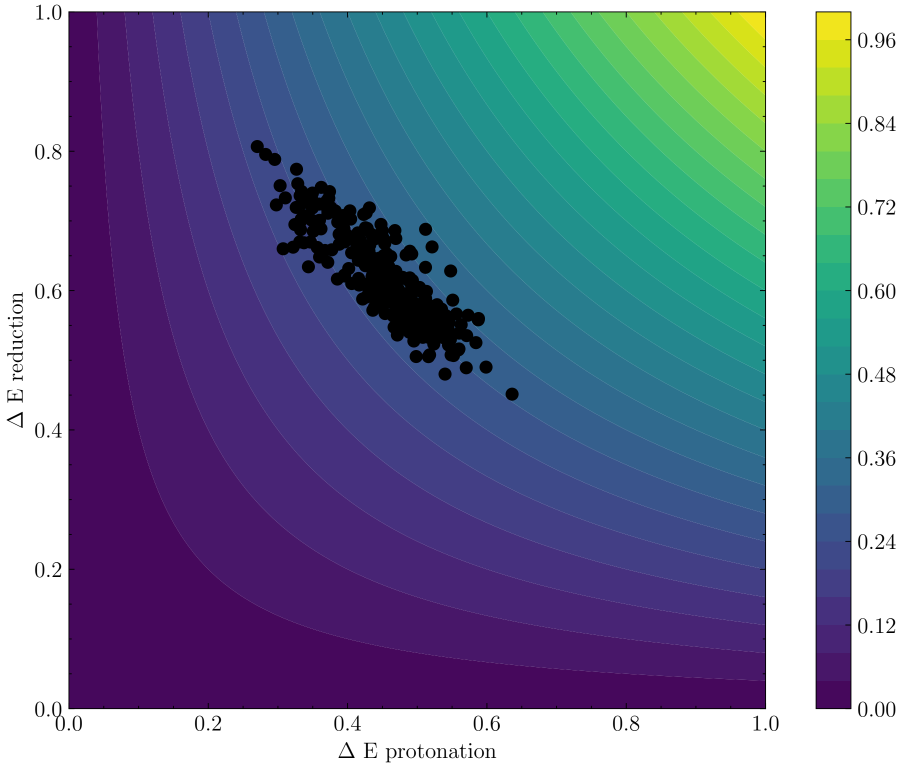

(a)

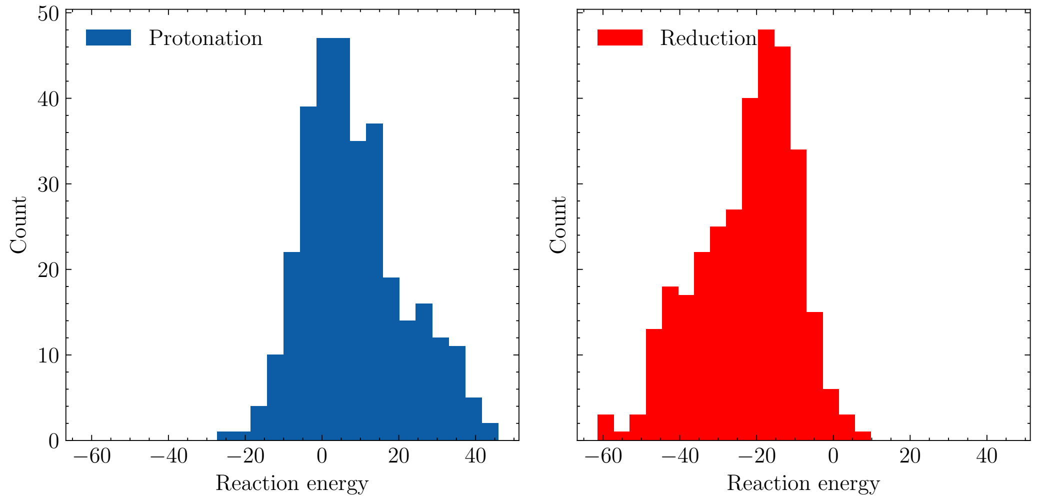

(b)

Figure S3: **(a)** Score surface of molecules obtained after conformer search for 1 neutral ligand molecules. The energies are normalized and without SA score modification. (**b**) Distribution of calculated energies.

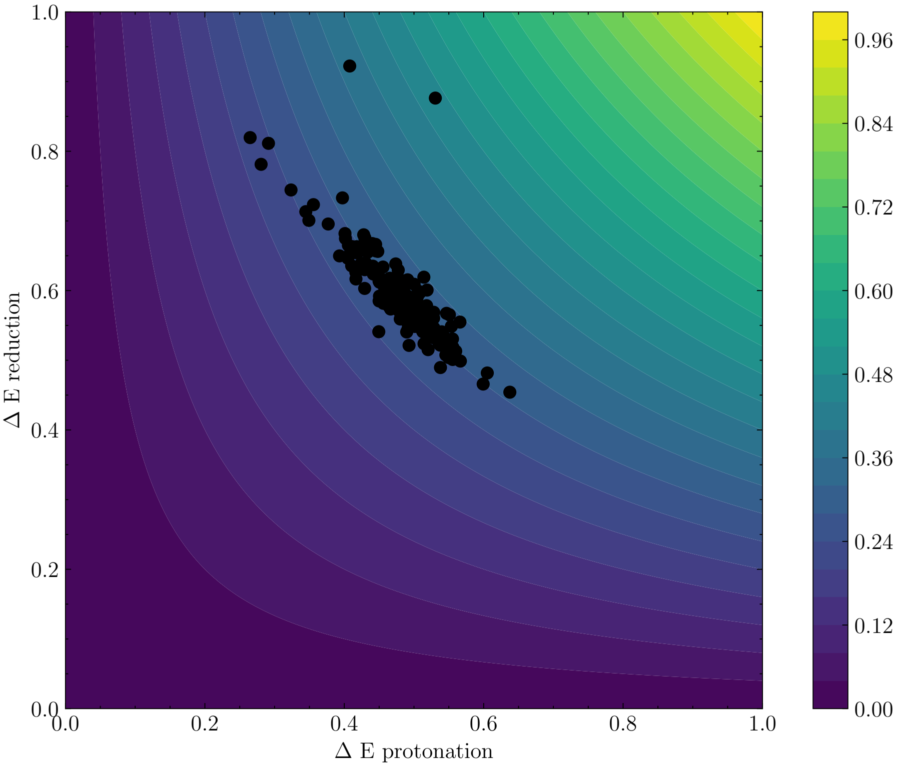

(a)

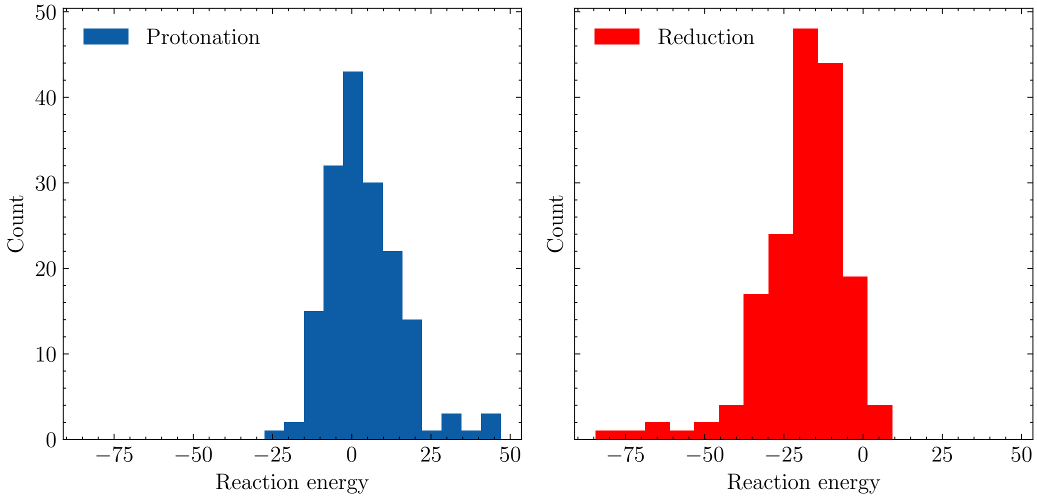

(b)

Figure S4: **(a)** Score surface of molecules obtained after conformer search for 2 neutral ligand molecules. The energies are normalized and without SA score modification. **(b)** Distribution of calculated energies.

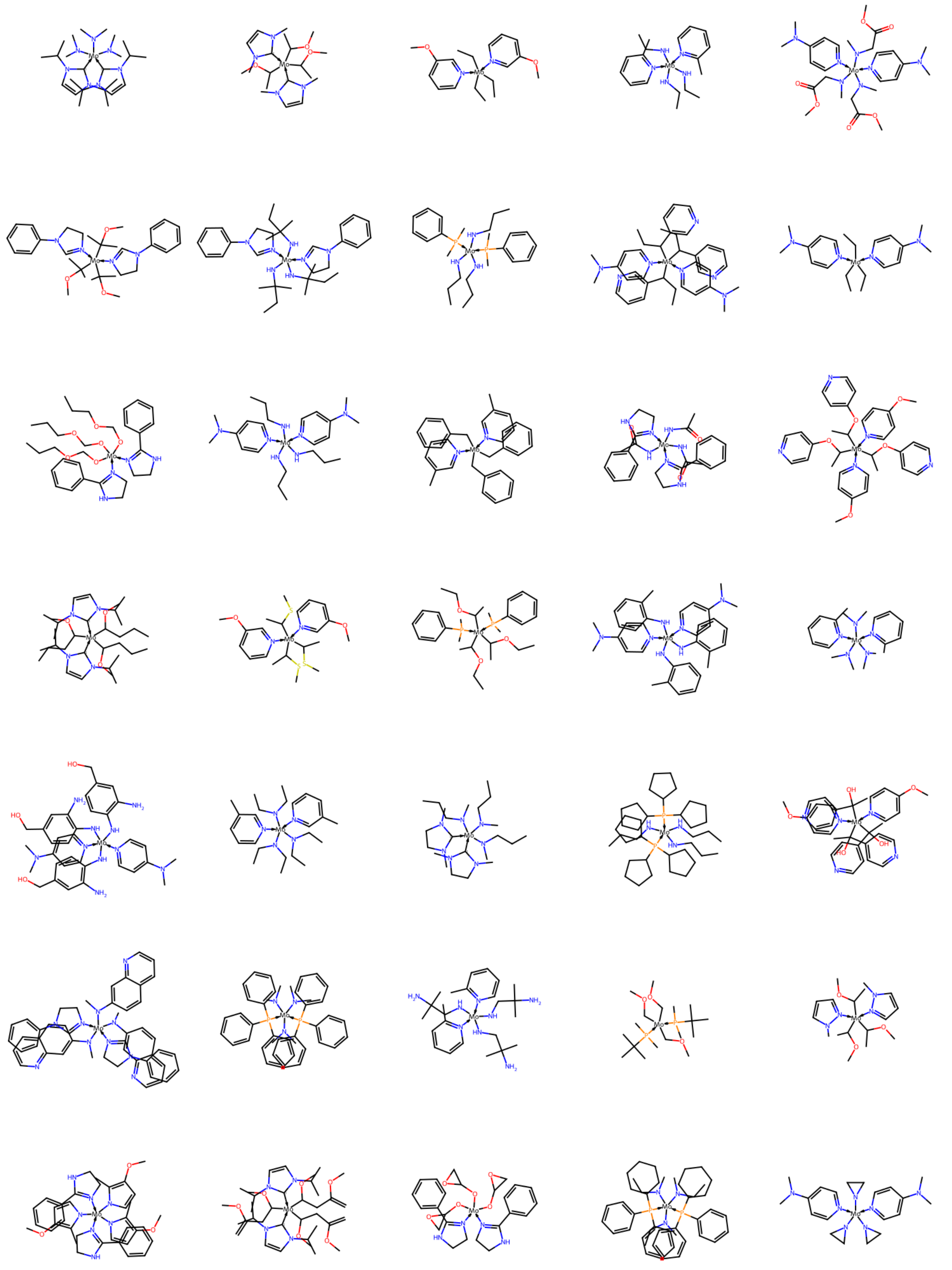

Figure S5: 40 molecules picked at random from the final population of molecules scored with 2 neutral ligand.

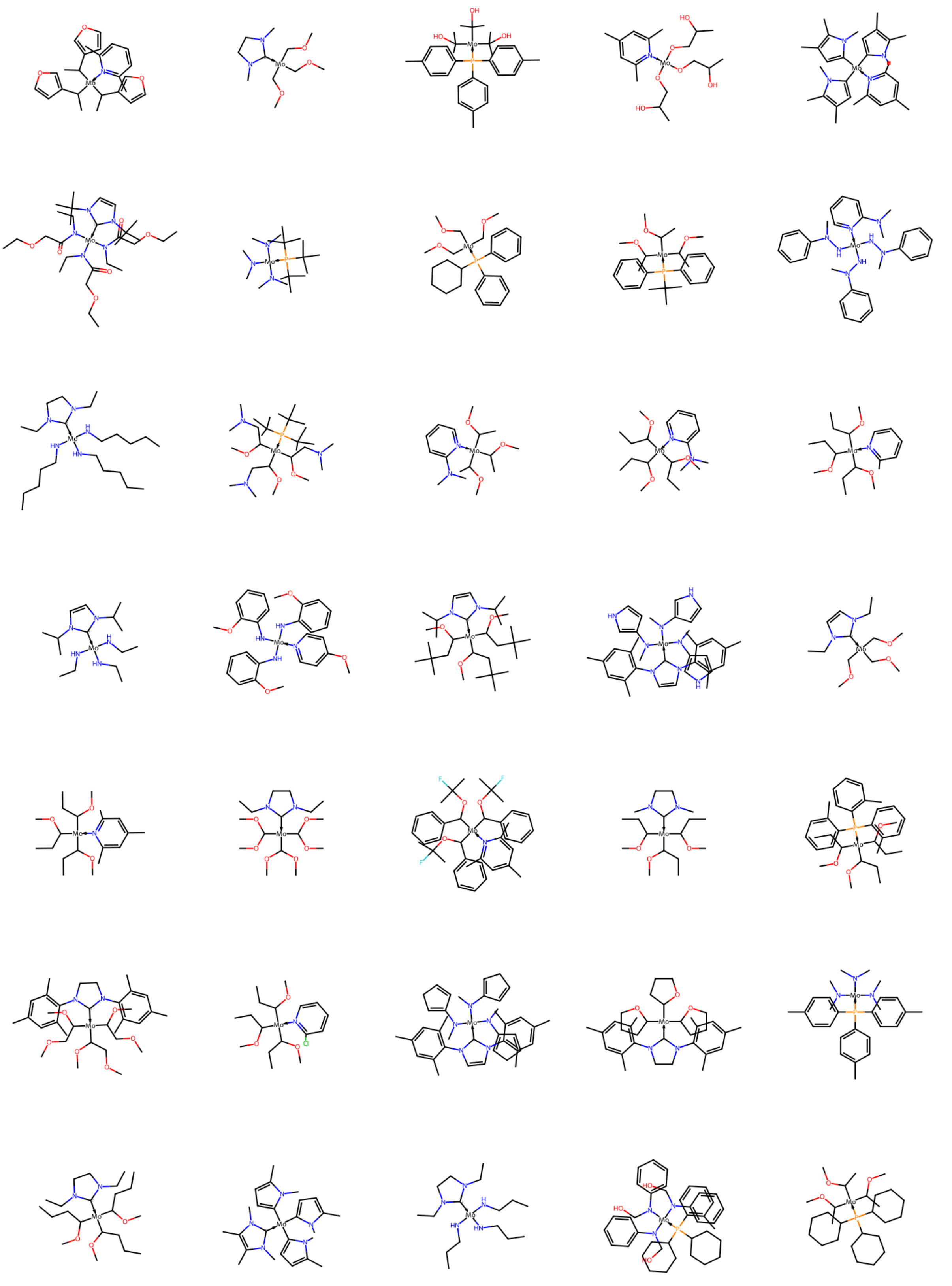

Figure S6: 40 molecules picked at random from the final population of molecules scored with 1 neutral ligand.

S2 SA score

In addition to the gaussian modifier suggested by Gao and Coley [1], we apply an extra gaussian modifier on the average of the SA score for the ligands in a catalyst. The form of this gaussian is visualized in figure S7. This modifier maps the upper range of values for the SA score into a smaller range on the y-axis. In this way, molecules in the upper range of average SA scores would need less improvement in reaction energy scores in order to survive throughout populations allowing for more evolutionary flexibility. Equation (S1) shows the form of the Gao and Coley [1] modifier and equation (S2) shows our extra modifier. Equation (S3) shows the final equation we use to obtain the final SA score.

$$\mathbf{SA}_{modified}(\mathrm{x}) = \exp\left(-\frac{1}{2}\left(\frac{x - 2.230044}{0.6526308}\right)^2\right) \tag{S1}$$

$$\mathbf{Extra}_{modifier}(\mathrm{x}) = \exp\left(-\frac{1}{2}\left(\frac{x - 1}{0.8}\right)^2\right) \tag{S2}$$

$$\mathrm{SA}_{score} = \mathbf{Extra}_{modifier}\left(\frac{3 \cdot \mathbf{SA}_{modified}(\mathrm{L}_1) + (1 \text{ or } 2) \cdot \mathbf{SA}_{modified}(\mathrm{L}_2)}{2}\right) \tag{S3}$$

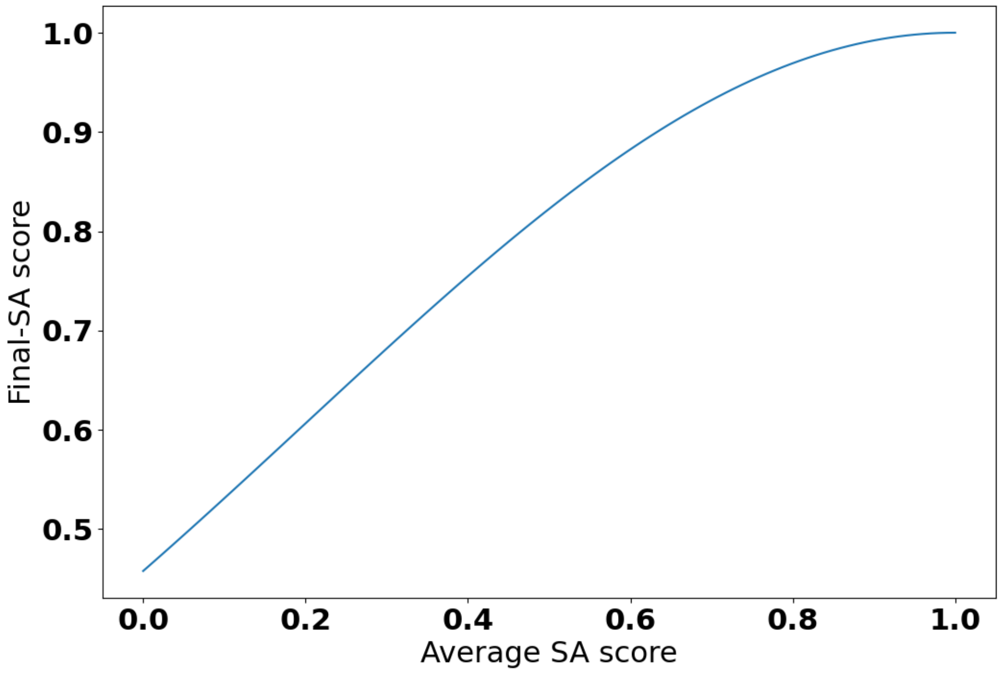

Figure S7: SA_{score} using our extra gaussian modifier. Gaussian constants are $\mu = 1$, $\sigma = 0.8$.

S3 Scoring surface

As discussed in the main text, the chosen method of combining scores was by multiplication of normalized reaction scores. Figure S8 shows two experiment GA runs with different

scoring objectives. The generations with addition of reaction energies is observed to be too flexible in both dimensions. Optimizing towards one reaction energy can significantly improve the score. As the first reduction is a heavily exergonic reaction for the schrock cycle, the GA could easily optimize towards ligands that improved mostly on this reduction reaction. The population moves almost exclusively upwards along the axis of reduction as generations increase. On the other hand (left in Figure S8), we have the minimization of the highest reaction energy which leads to a clear improvement of the protonation energy on the x-axis. The first reduction is usually highly exothermic, effectively meaning that this objective was likely only optimizing on the protonation energy.

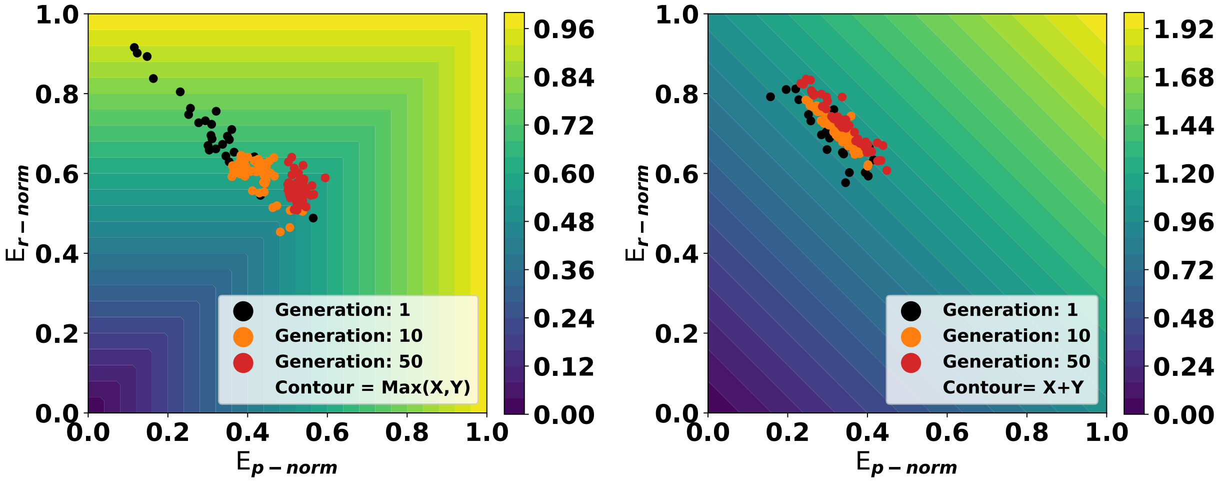

Figure S8: Example GA runs with two different scoring objectives. **Left)** Minimize the highest reaction energy. E.g maximize the lowest normalized reaction energy. **Right)** Maximize the sum of the normalized reaction energies

Figure S9 shows the result of a GA run where the starting population was comprised of the top 10 molecules from five different 50 generation runs. The population score improvement is noticeable from generation 1 to 10, but quickly converges and minor improvement is observed from generation 10 to 20. The improvement gained from the extra generations is minor and computation time could be better spent using a variety of non optimized starting populations.

S4 Crossover

A detailed illustration of the crossover procedure in the GA is shown in Figure S10. There are several random selection steps in the procedure, and unless otherwise specified a random selection has equal probability for each option. The first step of the crossover procedure is to select the catalyst and ligand we want to operate on. To do this, we first select Cat 1 or Cat 2 and then we select either the anionic (L_1) or the neutral (L_2) ligand in this catalyst. Then we can do one of two types of crossover on the chosen ligand. There is a 20% probability of simply performing a switch of the selected ligand with the ligand of the same type in the other catalyst. There is a 80% probability of performing ligand crossover between the selected ligand and the ligand of the same type in the other catalyst. The bottom of figure S10 shows an example in both cases where Cat 1 has been selected together with the anionic ligand L_1.

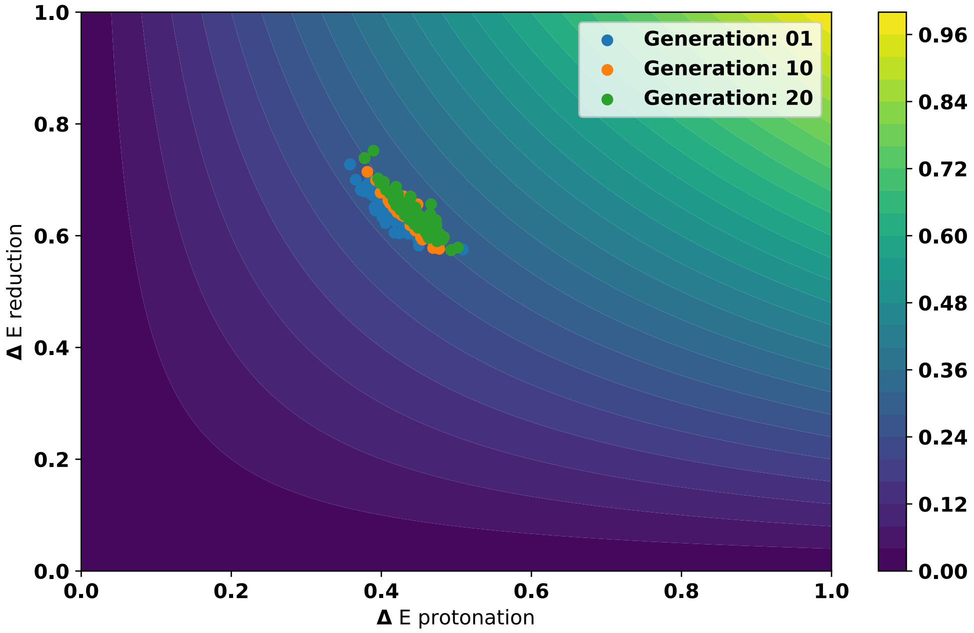

Figure S9: GA run with 50 molecules with a starting population obtained by combining the top 10 molecules from 5 separate GA runs. The energies are normalized

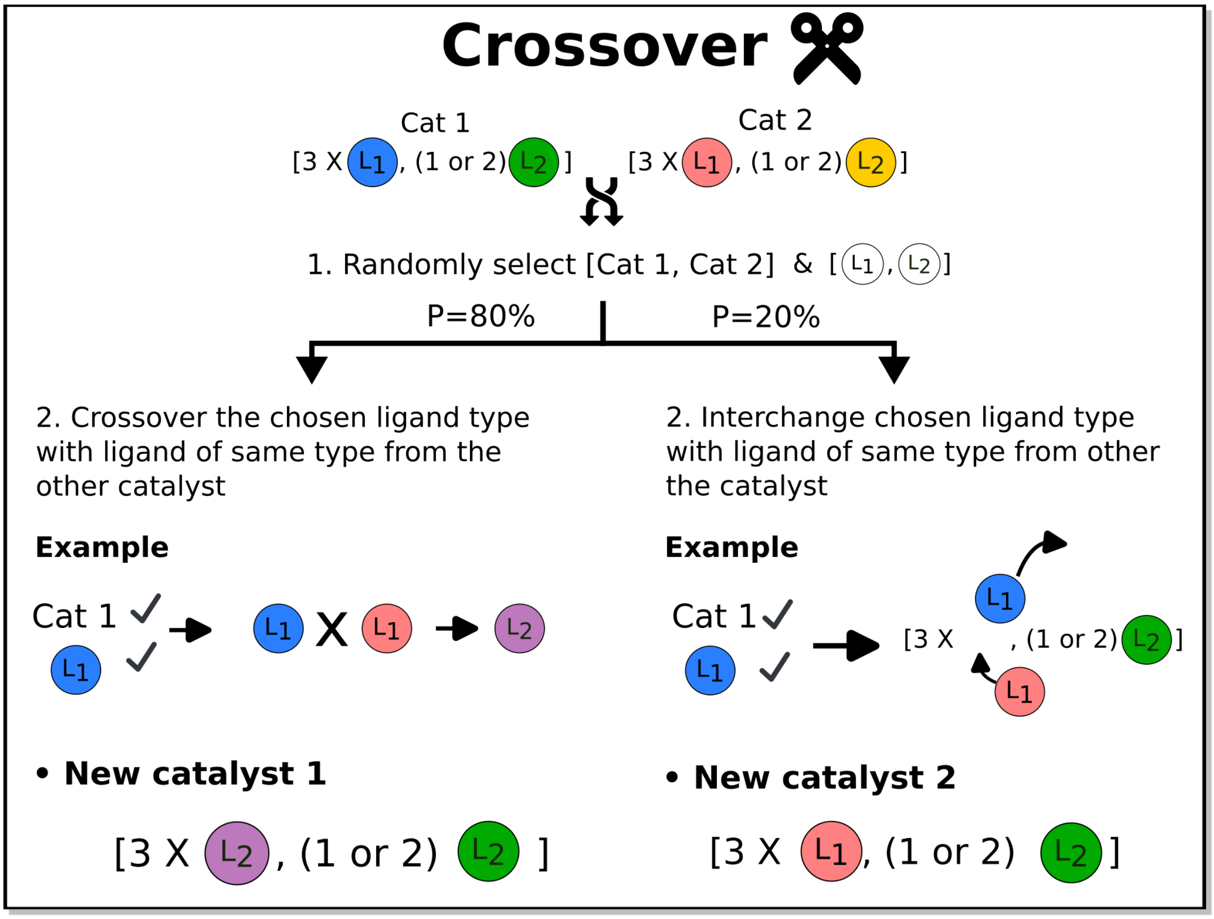

Figure S10: Illustration of the crossover operations in the GA.

S5 DFT calculations

S5.1 Schrock catalyst reference

The DFT results on all the Schrock catalyst reference intermediates and proton/electron donors are identical to the ones presented in our previous work[2].

S5.2 DFT methods

We apply identical DFT methods to previous approaches [2, 3]. ORCA 5 software [3] was utilized for all DFT computations. The PBE functional [4] is used for the screening process together with a relativistic basis set ZORA-def2-TZVP [5, 6]. For dispersion we use D3BJ [7, 8]. For the final energy refinement we apply B3LYP [9–11]/ZORA-def2-TZVP/D3BJ. The Split-RI-J [12] approximation was applied in PBE calculations, whereas RIJCOSX [13, 14] approximation was utilized in B3LYP calculations. The zeroth order regular approximation (ZORA [15]) was used to address relativistic effects. Finally, we use the CPCM [16] solvent model with $\epsilon = 1.844$.

S5.3 Errors for optimization of full cycles

Nitrogen fixation is an inherently difficult reaction. It was therefore found difficult to obtain intermediates for the whole catalytic cycle and in particular the catalysts with 1 neutral ligand proved difficult. Figure S11 shows 3 examples of intermediates where the catalyst has changed its configuration during DFT optimization. For catalysts with 1 and 2 neutral ligand configurations it was also observed that sometimes the incoming proton would move to a coordinating amide instead of the NxHx moiety, an example of this is shown in Figure S12.

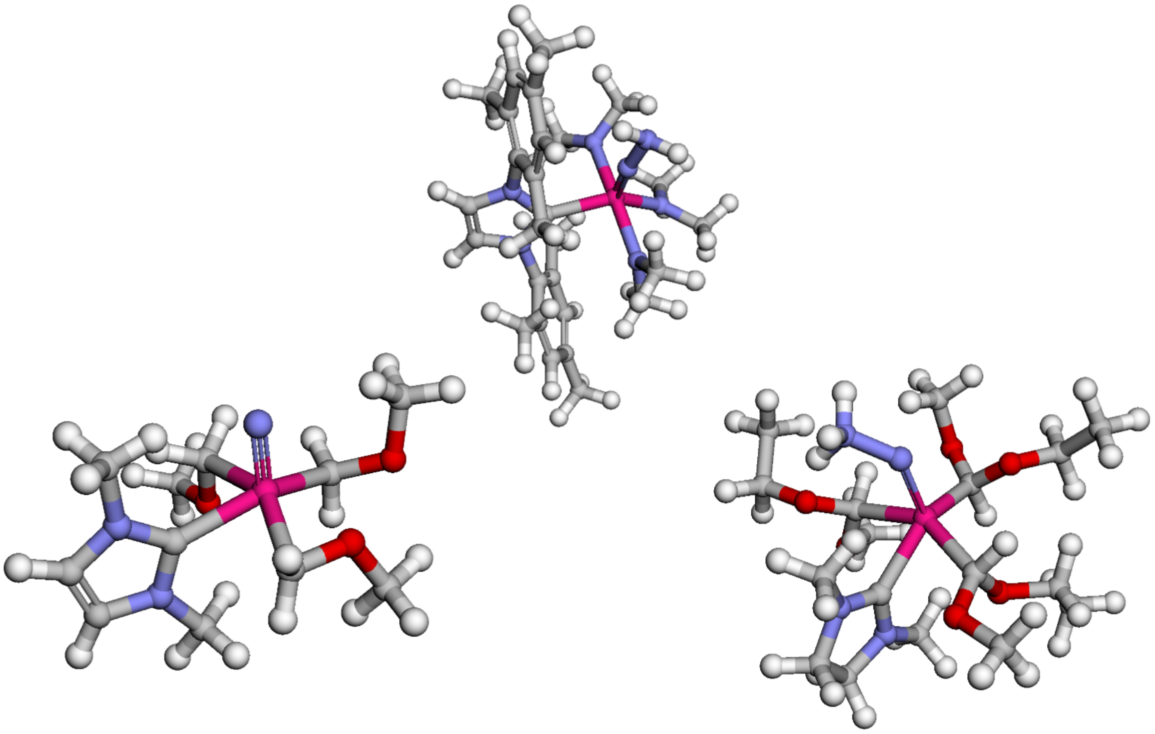

Figure S11: Examples of converged def2-TZVP/PBE structures for the catalysts with 1 neutral ligand in the trigonal bipyramidal configuration.

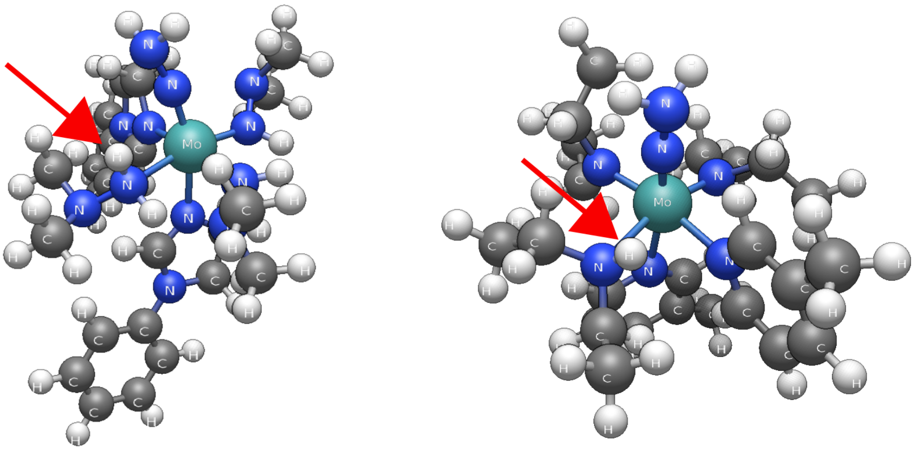

Cat1 **Cat2**

Figure S12: Illustration of protonated amide for the Mo$-N_2N_3^+$ intermediates for **Cat1** and **Cat2** of the 2 neutral ligand catalysts.

Top catalysts with 1 neutral ligand

The top 10 scoring catalysts for the catalysts with 1 neutral ligand is shown in Figure S13.

Catalyst 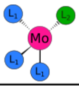	L_1	L_2	Score	$[E_p,E_r]$	Max(ΔE_{cat}-ΔE_{ref})
**Cat1**	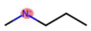	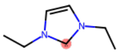	0.317	[-2.13,-24.13]	15.94
**Cat2**	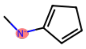	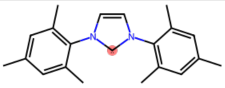	0.316	[-2.45,-23.33]	9.05
**Cat3**	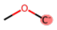	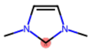	0.295	[-0.40,-17.55]	16.64
**Cat4**	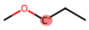	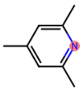	0.283	[4.42,-18.28]	13.92
**Cat5**	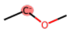	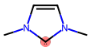	0.278	[5.38,-17.33]	18.85
**Cat6**	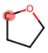	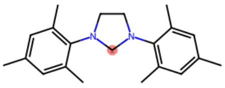	0.271	[3.58,-12.37]	15.7
**Cat7**	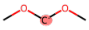	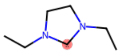	0.271	[5.83,-15.04]	15.51
**Cat8**	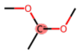	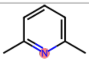	0.271	[8.62,-18.54]	8.76
**Cat9**	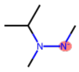	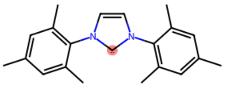	0.268	[-0.03,-7.14]	23.4
**Cat10**	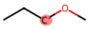	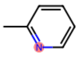	0.262	[9.54,-15.93]	9.45

Figure S13: Top 15 molecules after the final B3LYP characterization. Every catalyst is in a trigonal bipyramidal coordination geometry with $3 \cdot L_1$ and $1 \cdot L_2$. The score column contains the score based on the multiplication of normalized electronic reaction energies. The last column denotes the largest increase in reaction energy relative to the reference catalyst across the catalytic cycle.

S6 Linear scaling relations

The linear scaling relation for the 36 catalysts used to construct the volcano plot is shown in Figure S14. We excluded outliers by Chauvenets criterion with a cutoff of 5%.

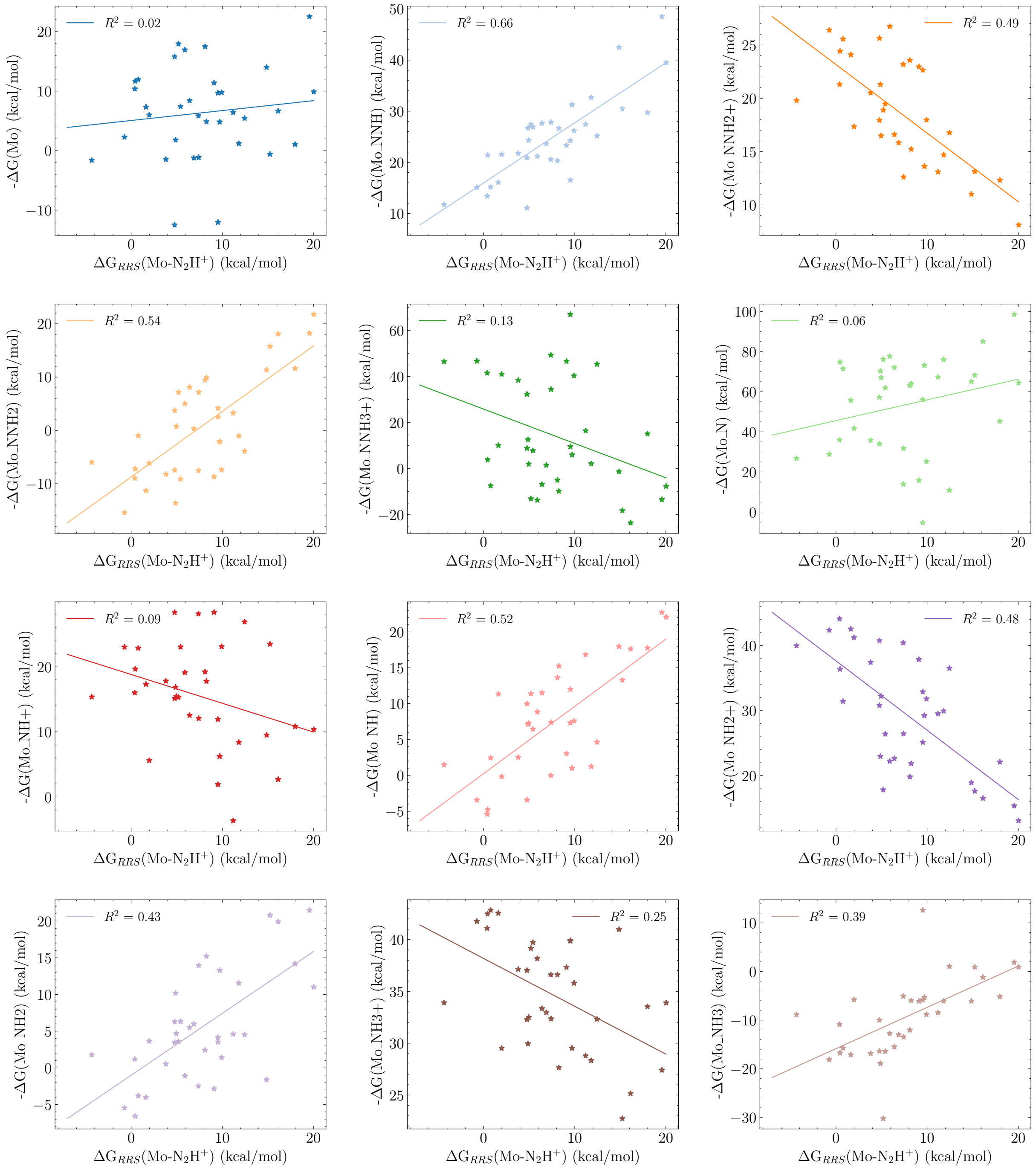

Figure S14: Linear scaling relationships based on 36 catalysts with B3LYP refined energies. R^2 indicates the coefficient of determination.

S7 Anionic carbons

The tmQMg-L dataset includes 5210 monodentates [17] that all have designated charge and coordinating atoms. We use the xyz2mol[1] package to obtain mol objects for each monodentate ligand. Then, using the coordinating atom information from the dataset, we search for all monodentates where a carbon is the coordinating atom and where it has a charge of -1. This gives us a total of 327 anionic ligands with an anionic carbon as coordinating atom. This number is only an estimate as 223 monodentate ligands fail the xyz2mol procedure. Additionally there can be incorrect overall ligands charges in the dataset which adds additional error to the estimate. Nevertheless, anionic carbons are existing in experimentally verified TM-complexes and therefore supports our approach to use such coordinating ligands for the GA search.

S8 Investigate ligand contributions to protonation energy

Figures S15, S16 and S17 contain the results from the ligand substitution study. Figure S18 shows an example of how this substitution looks for the 3D structures, using **Cat1** as example.

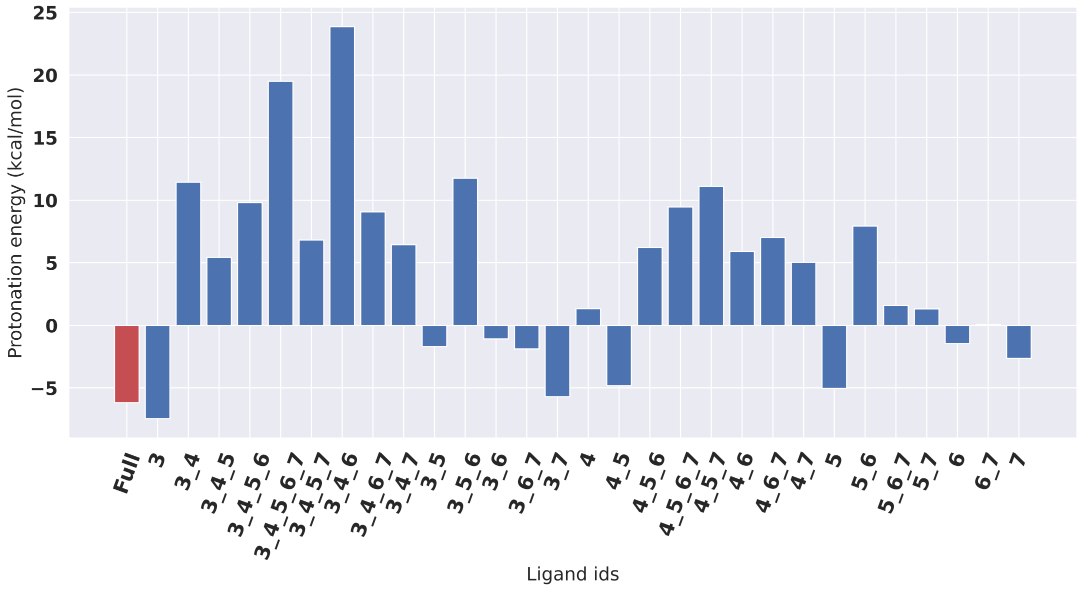

Figure S15: Ligand substitution energies for **Cat1**. The labels on the x-axis indicate the label of all ligands removed for each protonation energy calculation.

[1]https://github.com/jensengroup/xyz2mol

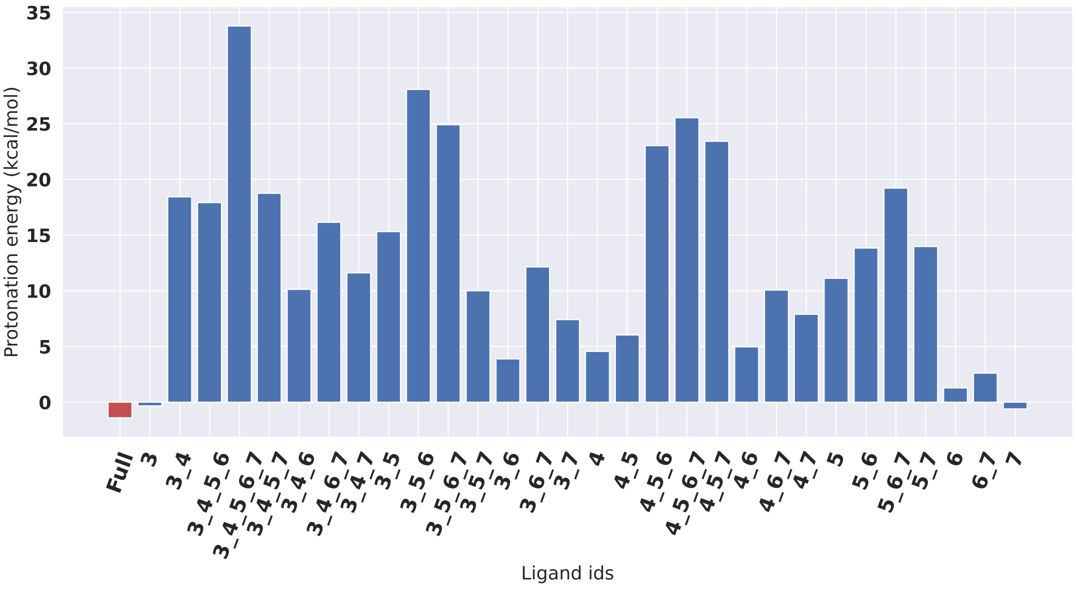

Figure S16: Ligand substitution energies for **Cat2**. The labels on the x-axis indicate the label of all ligands removed for each protonation energy calculation.

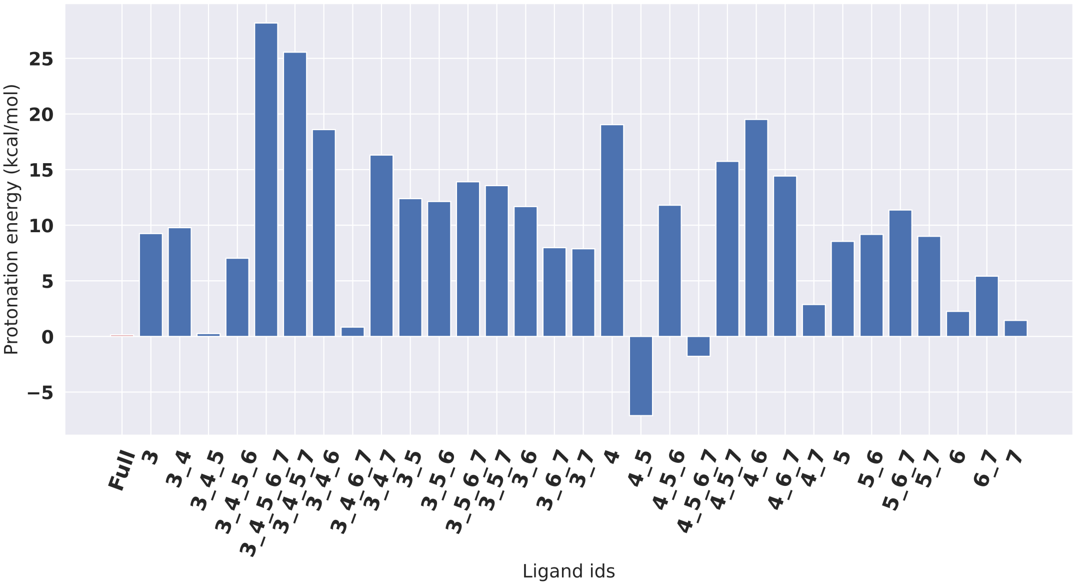

Figure S17: Ligand substitution energies for **Cat3**. The labels on the x-axis indicate the label of all ligands removed for each protonation energy calculation.

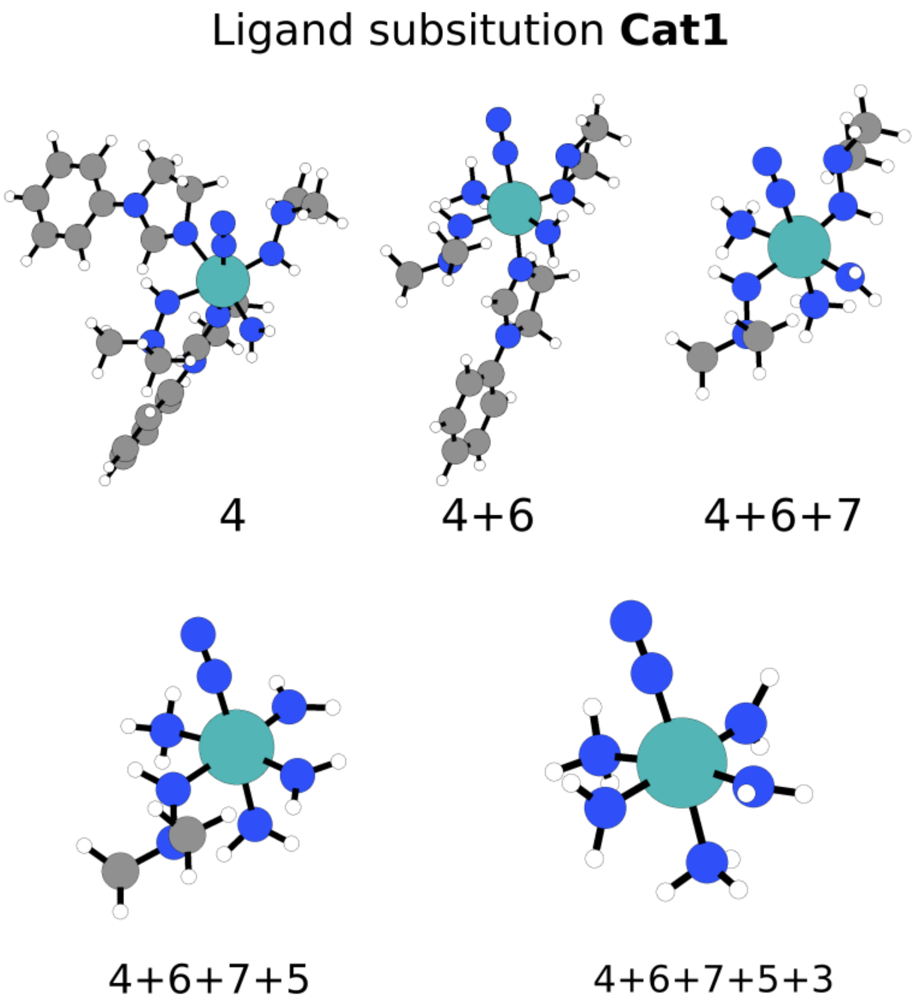

Figure S18: Example of the ligand substitution experiment for **Cat1** shown for the **Mo-N2** intermediate.

References

[1] W. Gao, C. W. Coley, *J. Chem. Inf. Model.* **2020**, *60*, 5714–5723.

[2] M. Strandgaard, J. Seumer, B. Benediktsson, A. Bhowmik, T. Vegge, J. H. Jensen, *PeerJ Phy. Chem.* **2023**, *5*, e30.

[3] F. Neese, *Wiley Interdiscip. Rev. Comput. Mol. Sci.* **2022**, *12*.

[4] J. P. Perdew, K. Burke, M. Ernzerhof, *Phys. Rev. Lett.* **1996**, *77*, 3865–3868.

[5] F. Weigend, R. Ahlrichs, *Phys. Chem. Chem. Phys.* **2005**, *7*, 3297–3305.

[6] D. A. Pantazis, X.-Y. Chen, C. R. Landis, F. Neese, *J. Chem. Theory Comput.* **2008**, *4*, 908–919.

[7] S. Grimme, J. Antony, S. Ehrlich, H. Krieg, *J. Chem. Phys.* **2010**, *132*, 154104.

[8] S. Grimme, S. Ehrlich, L. Goerigk, *J. Comput. Chem.* **2011**, *32*, 1456–1465.

[9] A. D. Becke, *J. Chem. Phys.* **1993**, *98*, 5648–5652.

[10] A. D. Becke, *Phys. Rev. A Gen. Phys.* **1988**, *38*, 3098–3100.

[11] C. Lee, W. Yang, R. G. Parr, *Phys. Rev. B Condens. Matter* **1988**, *37*, 785–789.

[12] F. Neese, *J. Comput. Chem.* **2003**, *24*, 1740–1747.

[13] F. Neese, F. Wennmohs, A. Hansen, U. Becker, *Chem. Phys.* **2009**, *356*, 98–109.

[14] R. Izsák, F. Neese, *J. Chem. Phys.* **2011**, *135*, 144105.

[15] E. van Lenthe, E. J. Baerends, J. G. Snijders, *J. Chem. Phys.* **1993**, *99*, 4597–4610.

[16] V. Barone, M. Cossi, *J. Phys. Chem. A* **1998**, *102*, 1995–2001.

[17] H. Kneiding, A. Nova, D. Balcells, *Nat Comput Sci* **2024**.
